# Mercury Monohalides as Ligands in Transition Metal Complexes

**DOI:** 10.3390/molecules30010145

**Published:** 2025-01-02

**Authors:** Matteo Busato, Jesús Castro, Domenico Piccolo, Marco Bortoluzzi

**Affiliations:** 1 Dipartimento di Chimica, Sapienza Università di Roma, P.le Aldo Moro 5, 00185 Rome, Italy; matteo.busato@uniroma1.it (M.B.); domenico.piccolo@unipd.it (D.P.); 2 Dipartimento di Scienze Molecolari e Nanosistemi, Università Ca’ Foscari Venezia, 30172 Mestre, Italy; 3 Departamento de Química Inorgánica, Facultade de Química, Universidade de Vigo, Edificio de Ciencias Experimentais, 36310 Vigo, Galicia, Spain; jesusc@uvigo.gal; 4 Dipartimento di Scienze Chimiche, Università di Padova, Via Marzolo 1, 35131 Padova, Italy; 5 CIRCC (Consorzio Universitario Reattività Chimica e Catalisi), Via Celso Ulpiani 27, 70126 Bari, Italy

**Keywords:** mercury, halides, transition metals, organometallic complexes, M-Hg bond

## Abstract

The main categories of transition metal–mercury heterometallic compounds are briefly summarized. The attention is focused on complexes and clusters where the {Hg-Y} fragment, where Y represents a halide atom, interacts with transition metals. Most of the structurally characterized derivatives are organometallic compounds where the transition metals belong to the Groups 6, 8, 9 and 10. More than one {Hg-Y} group can be present in the same compound, interacting with the same or with different transition metals. The main synthetic strategies are discussed, and structural data of representative compounds are reported. According to the isolobality with hydrogen, {Hg-Y} can form from one to three M-{Hg-Y} bonds, but further interactions can be present, such as mercurophilic and Hg···halide contacts. The formal oxidation state of mercury is sometimes ambiguous and thus {Hg-Y} can be considered as a Lewis acid or base on varying the transition metal fragment. Density functional theory calculations on selected Group 6 and Group 9 model compounds are provided in order to shed light on this aspect.

## 1. Transition Metal–Mercury Derivatives: General Aspects

Transition metal–mercury complexes were among the first compounds investigated in the field of direct metal–metal bonding. The fact that mercury can be readily attached to a large variety of transition metals has stimulated its use as a building block in the synthesis of mixed-metal clusters. Several examples of coordination and organometallic compounds where mercury formally behaves as a coordinating atom are thus present in the literature. As described in previous reviews [1,2,3,4,5], transition metal derivatives with mercury in the coordination sphere can be cataloged in few main categories.

**Figure 1:**
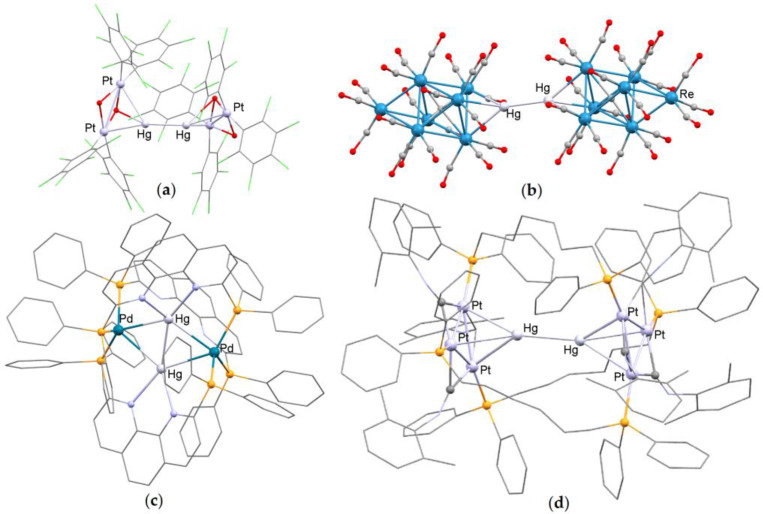
Molecular structures of (**a**) [Hg_2_{(C_6_Cl_5_)_2_Pt(μ-OH)_2_Pt(C_6_Cl_5_)_2_}_2_]^2−^ [6]; (**b**) [Hg_2_{Re_7_C(CO)_21_}_2_]^4−^ [7]; (**c**) [Hg_2_Pd_2_(P_2_phen)_3_]^2+^ [P_2_phen = 2,9-bis-(diphenylphosphino)-1,10-phenanthroline] [8]; (**d**) [Hg_2_{Pt_3_(RNC)_3_}_2_(diphos)_3_] [R = 2,6-Me_2_C_6_H_3_; diphos = 1,6-bis(diphenylphosphino)hexane] [9]. Color map: Hg, light grey; Pt, light violet; Re, blue; Pd, greenish blue; Cl, light green; P, orange; O, red; N, light blue; C, grey. Hydrogen atoms omitted.

The first possibility concerns species having general formula L_n_M_m_-Hg-Hg-M_m_L_n_, where the {Hg-Hg} group bonds two transition metal fragments. In most cases, the formal oxidation state of mercury is Hg(I). Recent examples are mixed-metal clusters having formulae [Hg_2_{(C_6_Cl_5_)_2_Pt(μ-OH)_2_Pt(C_6_Cl_5_)_2_}_2_]^2−^ [6] (Figure 1a), [Hg_2_{Re_7_C(CO)_21_}_2_]^4−^ [7] (Figure 1b), [Hg_2_M_2_(P_2_phen)_3_]^2+^ [M = Pd, Pt; P_2_phen = 2,9-bis-(diphenylphosphino)-1,10-phenanthroline] [8] (Figure 1c) and [Hg_2_{Pt_3_(RNC)_3_}_2_(diphos)_3_] [diphos = 1,5-bis(diphenylphosphino)pentane, 1,6-bis(diphenylphosphino)hexane; RNC = aromatic isocyanide] [9] (Figure 1d). The structure of [Hg_2_{(C_6_Cl_5_)_2_Pt(μ-OH)_2_Pt(C_6_Cl_5_)_2_}_2_]^2−^ is formally described as two [(C_6_Cl_5_)_2_Pt(μ-OH)_2_Pt(C_6_Cl_5_)_2_]^2−^ anions bridged by a [Hg_2_]^2+^ cation. Each mercury atom interacts with two platinum centers. The Hg-Hg distance is 2.552(3) Å, comparable with the Hg-Hg bond in mercurous nitrate, 2.5049(6) Å [10]. The Hg-Pt bond lengths are between 2.6629(10) and 2.9865(9) Å. [Hg_2_{Re_7_C(CO)_21_}_2_]^4−^ is composed of two carbidoheptarhenate clusters linked by a [Hg_2_]^2+^ cation, with Hg-Hg distance equal to 2.610(4) Å. The six Hg-Re bonds are in the 2.911(3)–2.965(3) Å range. In the Group 10 clusters [Hg_2_M_2_(P_2_phen)_3_]^2+^, the phosphine ligands coordinate the zero-valent M centers that interact with a [Hg_2_]^2+^ cation [Hg-Pd 2.7419(5)–2.7960(5) Å, Hg-Pt 2.7823(5)–2.8447(6) Å]. [Hg_2_]^2+^ is also coordinated by the nitrogen atoms and exhibits Hg-Hg bond lengths comprised between 2.6881(4) Å [M = Pd] and 2.7362(6) Å [M = Pt]. In the [Hg_2_{Pt_3_(RNC)_3_}_2_(diphos)_3_] clusters, the diphosphines bridge two {Pt_3_(μ-RNC)_3_} triangles, forming a cage where two mercury atoms are enclosed. The Hg-Hg distances are between 2.826(2) and 2.8424(2) Å, while the Hg-Pt bonds are in the 2.858(3)–2.980(3) range. Different from the previous examples, the mercury centers are considered as zero-valent, which highlights the sometimes ambiguous oxidation state of mercury in transition metal derivatives.

**Figure 2:**
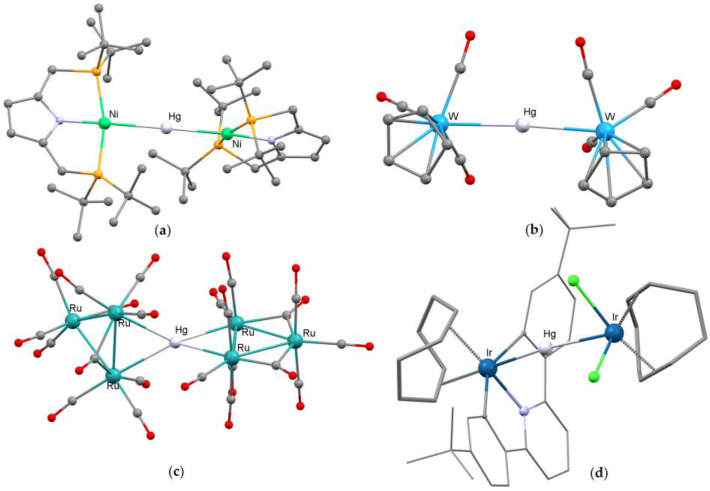
Molecular structures of (**a**) [Hg{Ni(PNP)}_2_] [PNP = 2,5-bis(di-*tert*-butylphosphanylmethyl)-pyrrolate] [11]; (**b**) [Hg{W(η^5^-C_5_H_5_)(CO)_3_}_2_] [12]; (**c**) [HgRu_6_(CO)_22_]^2−^ [15]; (**d**) [Ir(C^N^C)(COD)HgIrCl_2_(COD)] [H_2_C^N^C = 2,6-bis(4-*tert*-butylphenyl)pyridine; COD = 1,5-cyclooctadiene] [16]. Color map: Hg, light grey; Ir, dark blue; W, blue; Ru, bluish green; Ni, green; Cl, light green; P, orange; O, red; N, light blue; C, grey. Hydrogen atoms omitted.

In another group of compounds, a single mercury atom can behave as bridge between two or more transition metals. Selected examples are the trinuclear derivatives [Hg{Ni(PNP)}_2_] [PNP = pyrrolate-based pincer ligand] [11] (Figure 2a) and [Hg{W(η^5^-C_5_H_5_)(CO)_3_}_2_] [12] (Figure 2b). The Hg-Ni bonds in [Hg{Ni(PNP)}_2_] are 2.6488(4) and 2.6491(4) Å, while Hg-W distance of 2.7513(3) Å was measured for the two mercury-wolfram bonds in [Hg{W(η^5^-C_5_H_5_)(CO)_3_}_2_]. The mercury center can also join transition metal clusters. The structure of [Hg{Ru_6_C(CO)_16_}_2_]^2−^ is composed of two carbidohexaruthenate fragments, each one forming two Hg-Ru bonds falling in the 2.787(2)–2.902(1) Å range [13]. In [Hg{Ru_3_(μ_3_-ampy)(CO)_9_}_2_] [Hampy = 2-amino-6-methylpyridine], two trinuclear ruthenium fragments are connected by a single mercury atom, forming four Ru-Hg bonds with lengths comprised between 2.839(1) and 2.859(1) Å [14]. Ruthenium clusters having formulae [HgRu_6_(CO)_22_]^2−^ (Figure 2c), [Hg_2_Ru_7_(CO)_26_]^2−^, [Hg_3_Ru_8_(CO)_30_]^2−^ and [Hg_4_Ru_10_(CO)_32_]^4−^ were obtained by reacting [HRu_3_(CO)_11_]^−^ or [HRu_4_(CO)_12_]^3−^ with mercury(II) acetate or chloride [15]. The Hg-Ru bonds vary from 2.6726(13) to 2.9079(10) Å.

Mercury can also bridge transition metals with different coordination spheres. Recent compounds of this type are the polynuclear clusters [Ir_2_Cl_2_(μ-Cl)_2_(COD)_2_{HgIrCl(κ^2^*C,N*-HC^N^C)(COD)}_2_] and [Ir(C^N^C)(COD)HgIrCl_2_(COD)] (Figure 2d) [H_2_C^N^C = 2,6-bis(4-*tert*-butylphenyl)pyridine; COD = 1,5-cyclooctadiene]. The first structure can be formally described considering a divalent mercury center between a dinuclear [Ir_2_Cl_2_(μ-Cl)_2_(COD)_2_]^2−^ cluster and a [IrCl(κ^2^*C,N*-HC^N^C)(COD)]^−^ complex [Hg-Ir bonds 2.6314(3) and 2.5829(3) Å], while in the second compound Hg(II) joins the [Ir(C^N^C)(COD)]^−^ and [IrCl_2_(COD)]^−^ complexes, with Hg-Ir bond lengths equal to 2.5841(3) and 2.6656(3) Å [16]. Finally, mercury can bridge different transition metals. [Hg_2_{Co_6_C(CO)_12_}{W(η^5^-C_5_H_5_)(CO)_3_}_2_]^2−^ [17] is composed by a carbidohexacobaltate cluster connected to two {W(η^5^-C_5_H_5_)(CO)_3_} fragments by means of two mercury centers, each one forming three Hg-Co bonds [2.711(2)–2.7261(19) Å] and a Hg-W bond [2.781(2) Å]. In [{Re(CO)_4_Mo(η^5^-C_5_H_5_)(CO)_2_(μ-PCy_2_)}Hg{W(η^5^-C_5_H_5_)(CO)_3_}] [Cy = cyclohexyl], mercury joins a dinuclear Re-Mo cluster with a {W(η^5^-C_5_H_5_)(CO)_3_} fragment [18]. The Hg-M bonds are equal to 2.790(1) Å [M = Re], 2.940(1) Å [M = Mo] and 2.780(1) Å [M = W].

**Figure 3:**
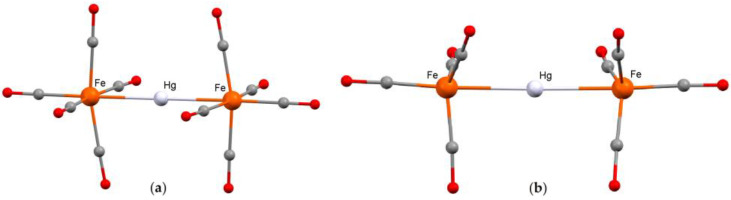
Molecular structures of (**a**) [Hg{Fe(CO)_5_}_2_]^2+^ [19]; (**b**) [Hg{Fe(CO)_4_}_2_]^2−^ [20]. Color map: Hg, light grey; Fe, reddish orange; O, red; C, grey.

The definition of the most correct formal oxidation state of mercury in heteropolymetallic species is generally not straightforward. Recently, Frenking, Malischewski and co-workers investigated the [Hg{Fe(CO)_5_}_2_]^2+^ (Figure 3a) and [Hg{Fe(CO)_4_}_2_]^2−^ (Figure 3b) trinuclear species, characterized by Hg-Fe distances equal to 2.5745(7) and 2.546(2) Å, respectively [19,20,21]. According to the energy decomposition analysis with natural orbitals for chemical valence, in both cases the mercury center is best described as Hg(0) instead of Hg(II), thus behaving as a σ-donor toward the iron fragments.

**Figure 4:**
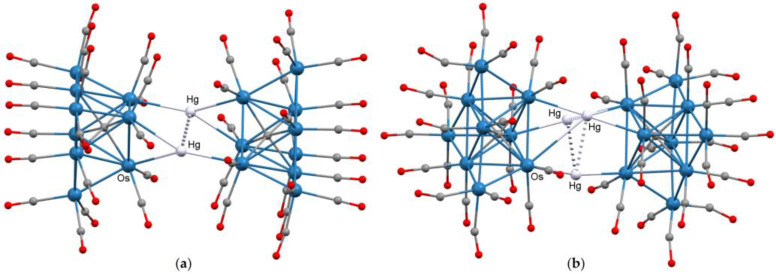
Molecular structures of (**a**) [Hg_2_{Os_9_(C)(CO)_21_}_2_]^4−^ [26]; (**b**) [Hg_3_{Os_9_(C)(CO)_21_}_2_]^2−^ [27]. Color map: Hg, light grey; Os, blue; O, red; C, grey.

The description of clusters containing more than one mercury atom in the structure must also account for mercurophilicity, i.e., the metallophilic interactions possibly occurring among mercury centers, in particular if belonging to the same compound [15,22,23,24,25]. Despite the fact that the examples of mercurophilic interactions in the literature are less abundant than those concerning aurophilicity, the possibility of closed-shell Hg(II)···Hg(II) interactions should be taken into account when the Hg···Hg distance is in the range of the Van der Waals contact (about 3.5 Å) or lower. For instance, a dimeric [Hg_2_]^4+^ unit with sub van der Waals Hg(II)···Hg(II) distance, 2.820(3) Å, is present in [Hg_2_{Os_9_(C)(CO)_21_}_2_]^4−^ (Figure 4a) [26]. Such a compound derives from the cluster [Hg_3_{Os_9_(C)(CO)_21_}_2_]^2−^ (Figure 4b), where a [Hg_3_]^6+^ triangular unit bridges two carbidoennaosmiate fragments. The Hg···Hg(II) distances in the trimercury fragment are comprised between 2.920(7) and 2.931(6) Å [27]. The shortest Os-Hg bond length measured for these clusters is 2.696(5) Å. As another example, the structure of the cation [Hg_8_{Ir(η^5^-C_5_Me_5_)(CO)}_6_] contains a central {Hg_4_} fragment with Hg···Hg distances between 2.982(2) and 3.0278(18) Å. Each mercury of {Hg_4_} bonds two iridium centers [28]. The compound also contains two {Hg_3_} triangles with Hg···Hg distances in the 2.962(2)–3.078(2) Å range.

**Figure 5:**
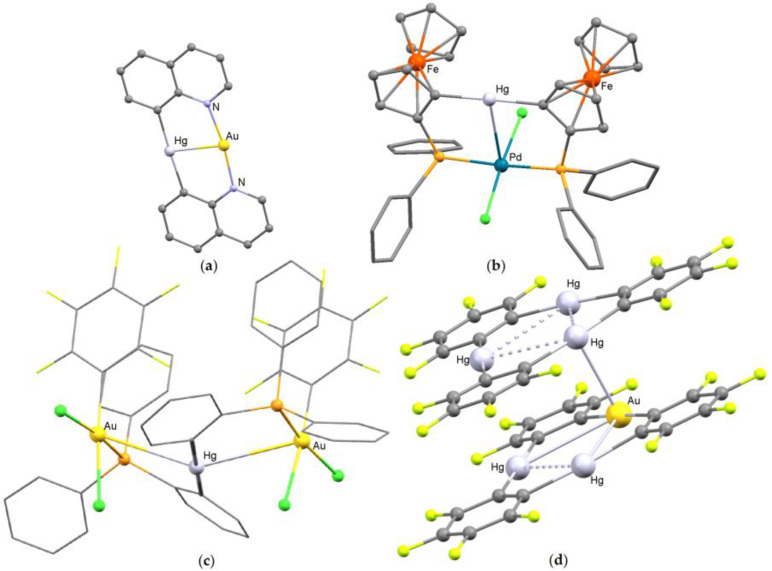
Molecular structures of (**a**) [Au{Hg(C_9_H_6_N)_2_}]^+^ [34]; (**b**) [PdCl_2_{Hg(C_5_H_3_PPh_2_-Fe-C_5_H_5_)_2_}] [36]; (**c**) [{AuCl_2_(C_6_F_5_)}_2_{Hg(C_6_H_4_PPh_2_)_2_}] [40]; (**d**) [{AuHg_2_(*o*-C_6_F_4_)_3_}{Hg_3_(*o*-C_6_F_4_)_3_}]^−^ [44]. Color map: Hg, light grey; Au, yellow; Pd, greenish blue; Fe, reddish orange; Cl, light green; P, orange; F, greenish yellow; C, grey. Hydrogen atoms omitted.

In the last category of compounds, mercury is bonded to one or two Y ligands and directly to a metal center, with the formation of species having general formula L_n_M_m_-HgY_y_. The Y ligands can have different nature, according to the typical coordination chemistry of mercury [29,30,31,32,33]. Given the noticeable stability of the Hg-C σ-bonds, transition metal organomercury derivatives were investigated in detail. Mercury is usually bonded to two C-donor fragments that are part of the ancillary ligands surrounding the transition metal. Very short metallophilic interactions between Hg(II) and either Pd(II) or a Group 11 M(I) center were recently observed using quinolin-8-yl fragments able to form Hg-C together with M-N bonds [34]. Figure 5a shows the molecular structure of the Au(I) derivative, characterized by Hg(II)···Au(I) distance equal to 2.596(3) Å. Organomercury-bridged diphosphines are another class of compounds able to form heteropolymetallic complexes with short metal–mercury interactions [35,36,37,38]. The structure of a Pd(II) derivative [Hg(II)···Pd(II) 2.9828(6) Å] is shown in Figure 5b. Two Hg···M interactions can be present in the same molecule if the diphosphine behaves as a bridging ligand, as occurs in the bis(rhodium) complex formed with the ligand (η^5^-C_5_H_5_)Fe(PPh_2_C_5_H_3_-Hg-C_5_H_3_PPh_2_)Fe(η^5^-C_5_H_5_) and in the Au(I) and Au(III) derivatives [{Au(Ar)}_2_{Hg(C_6_H_4_PPh_2_)_2_}] and [{AuCl_2_(Ar)}_2_{Hg(C_6_H_4_PPh_2_)_2_}] [Ar = halide-substituted aryls] (see for instance Figure 5c) [39,40]. The Hg···Au(I) distances are between 3.1222(3) and 3.1950(3) Å, while the Hg···Au(III) distances are longer, 3.3973(3) Å. As another example, the reaction of a Cu(I) precursor with a Hg_2_N_4_-donor macrocycle afforded a heterometallic species with intramolecular Hg···Cu [2.919(7)–2.921(7) Å] and intermolecular Hg···Hg [3.203(4) Å] metallophilic contacts [41,42]. Unsupported interactions between HgR_2_ (R = organometallic ligand) and Group 10 or Group 11 transition metal complexes were however reported [43,44,45]. For instance, in the anion [{AuHg_2_(*o*-C_6_F_4_)_3_}{Hg_3_(*o*-C_6_F_4_)_3_}]^−^ the two metallacycles are connected by a short Hg(II)···Au(I) contact, 3.097(2) Å, and the computed interaction energy is around 47.7 kcal mol^−1^ (Figure 5d).

## 2. Heterometallic Transition Metal Complexes with Mercury Dihalides

Divalent mercury halides are among the most common HgY_2_ compounds in the chemistry of mercury [46]. The interaction between transition metals in low oxidation state and mercury(II) halides was observed in a number of cases while studying the Lewis basicity of metal carbonyl complexes [47,48,49,50,51,52,53,54]. The synthetic approach was based on the direct reaction of a suitable transition metal carbonyl precursor with HgY_2_. Calorimetric measurements on the reaction between Group 6 transition metal carbonyl derivatives and mercury(II) halides in 1,2-dichloroethane solution indicated that the Hg-M bonds formed are at least as strong as the interactions of divalent mercury with conventional Lewis bases. In some cases, the Gibbs energy variation for the reaction is negative by about 7 kcal mol^−1^ [55]. Another reaction pathway involves the formal insertion of metallic mercury in the M-Y bond, even if such a reaction should be considered a redox process. For instance, dinuclear {Fe-HgI_2_} complexes with π-acceptor ligands surrounding the iron center were obtained by reacting [FeI_2_(CNR)_4_] [R = alkyl, aryl] with mercury in the presence of isocyanides and phosphines [56,57]. Yamamoto and co-workers isolated a product having formula [Ni(HgI_2_)(CNR)_4_] [R = 2,6-Me_2_C_6_H_3_] by reacting [NiI_2_(CNR)_2_] with mercury in the presence of isocyanide. Moreover, the authors assumed the formation of a transient [Ni(HgI_2_)(CNR)_2_] species while investigating the electrochemical behavior of [NiI_2_(CNR)_2_] with a mercury electrode [58].

The M-HgY_2_ interaction is generally described as a donation of electron density from the transition metal in a low oxidation state, behaving as Lewis base, to HgY_2_, which acts as Lewis acid. However, computational studies on [Ru(M’Cl_2_)(CO)_3_(PPh_2_py)_2_] [M’ = Zn, Cd, Hg; PPh_2_py = diphenyl-2-pyridylphosphine] revealed that such an assumption is correct for M’ = Zn, while the Ru←M’ back-donation is relevant for both M’ = Cd and M’ = Hg [59].

**Figure 6:**
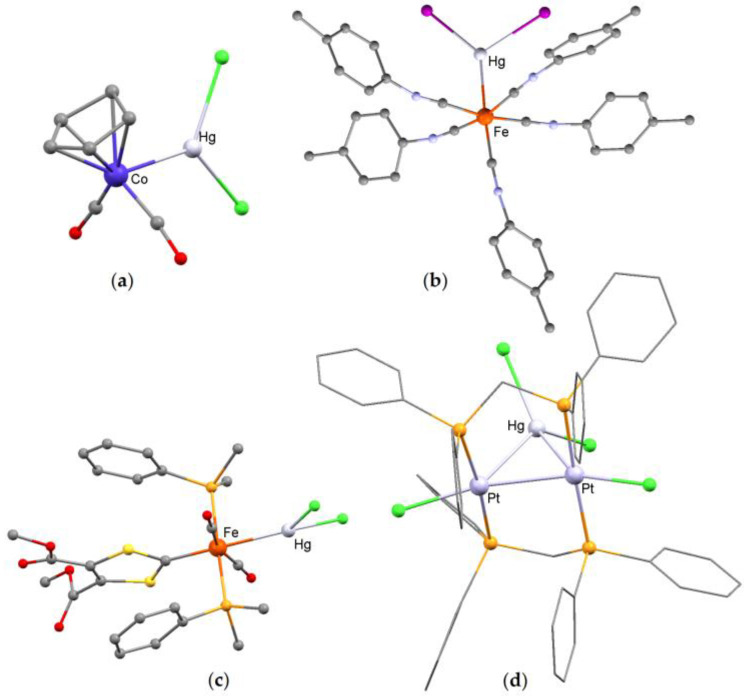
Molecular structures of (**a**) [Co(HgCl_2_)(η^5^-C_5_H_5_)(CO)_2_] [60]; (**b**) [Fe(HgI_2_){CN(4-MeC_6_H_4_)}_5_] [56]; (**c**) [Fe(HgCl_2_)(CO)_2_(PMe_2_Ph)_2_{CS_2_C_2_(CO_2_Me)_2_}] [62]; (**d**) [Pt_2_(HgCl_2_)Cl_2_(dppm)_2_] [dppm = bis(diphenylphosphino)methane] [64]. Color map: Hg, light grey; Pt, light violet; Co, blue; Fe, reddish orange; I, purple; Cl, light green; S, yellow; P, orange; O, red; C, grey. Hydrogen atoms omitted.

One of the earliest examples of M-HgY_2_ derivatives investigated by means of single-crystal X-ray diffraction is [Co(HgCl_2_)(η^5^-C_5_H_5_)(CO)_2_] (Figure 6a), showing a Hg-Co bond length equal to 2.578(4) Å. The compound was obtained from [Co(η^5^-C_5_H_5_)(CO)_2_] and HgCl_2_ [60]. It is worth noting that the use of the related isocyanide precursor [Co(η^5^-C_5_H_5_){CNC(O)C_6_H_5_}_2_] afforded a less stable heterobimetallic product [61]. Other species investigated by means of X-ray diffraction are [Fe(HgI_2_){CN(*p*-tolyl)}_5_] [Hg-Fe 2.551(1) Å] (Figure 6b) [56] and [Fe(HgCl_2_)(CO)_2_(PMe_2_Ph)_2_{CS_2_C_2_(CO_2_Me)_2_}] [Hg-Fe 2.546(1) Å] (Figure 6c) [62,63]. Structurally characterized examples of trinuclear metal complex showing bridging coordination mode for HgCl_2_ are [Pt_2_(HgCl_2_)Cl_2_(dppm)_2_] (Figure 6d) and [Rh_2_(HgCl_2_)(η^5^-C_5_H_5_)_2_(μ-dppm)(μ-CO)] [dppm = bis(diphenylphosphino)methane]. In the first species, the two Hg-Pt bond lengths are between 2.6991(8) and 2.7153(7) Å, contributing to the formation of an “A-frame” structure with HgCl_2_ at one vertex of the trimetallacycle [64]. Relevant parameters for the trinuclear rhodium derivative are Hg-Rh distances comprised between 2.692(1) and 2.744(2) Å and Hg-Cl bonds comprised between 2.534(3) and 2.581(3) Å [65].

HgY_2_ can also bond with transition metal clusters through the interaction with halides. For instance, in the structure of [Pt_2_(dppp)_2_{(μ_3_-Cl)_2_HgI_2_}] [dppp = 1,3-bis(diphenylphosphino)propane], HgI_2_ is connected to the chloro-ligands bridging the Pt centers, and only a weak Hg···Pt interaction [3.1744(4) Å] is present [66].

**Figure 7:**
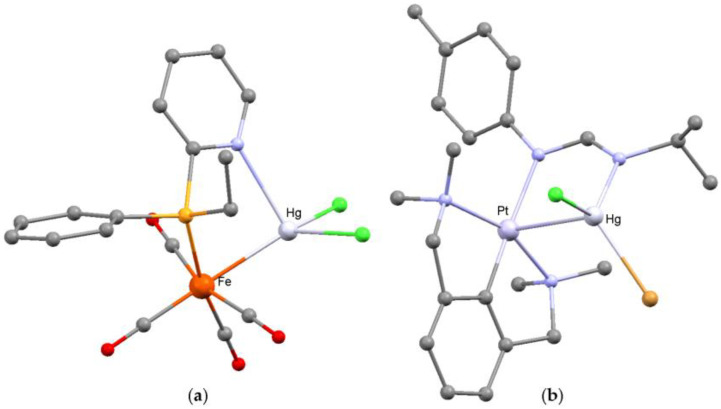
Molecular structures of (**a**) [Fe(HgCl_2_)(CO)_4_(PEtPhpy)] [PEtPhpy = 2-(ethylphenylphosphino)pyridine] [67]; (**b**) [Pt{2,6-(Me_2_NCH_2_)_2_C_6_H_3_}{μ-(*p*-tolyl)NC(H)N(*^i^*Pr)}HgBrCl] [72]. Color map: Hg, light grey; Pt, light violet; Fe, reddish orange; Br, dark orange; Cl, light green; P, orange; O, red; C, grey. Hydrogen atoms omitted.

The bond between the transition metal fragment and HgY_2_ can be enforced in the presence of suitable ancillary ligands. For instance, the complex [Fe(CO)_4_(PEtPhpy)] [PEtPhpy = 2-(ethylphenylphosphino)pyridine] undergoes an addition reaction with HgCl_2_ to afford the binuclear derivative [Fe(HgCl_2_)(CO)_4_(PEtPhpy)] (Figure 7a), where a Hg-Fe bond [2.608(1) Å] is present together with a Hg-N interaction [2.530(4) Å] [67]. In the compound [Fe(HgI_2_)(CO)_3_(PPh_2_py)_2_], obtained following the same approach, the interaction between HgI_2_ and the transition metal fragment is essentially due to the Hg-Fe bond [2.6780(2) Å], even if two weak mercury–nitrogen interactions are present, the shortest one with Hg-N distance equal to 2.658(2) Å [68]. In related complexes having the general formula [Fe(HgY_2_)(CO)_3_(Ph_2_Ppym)_2_] [Y = Cl, Br, I; Ph_2_Ppym = 2-(diphenylphosphino)pyrimidine], the Hg-Fe distances are comprised between 2.616(2) and 2.665(2) Å. In the case of Y = Cl, the mercury center weakly interacts with the nitrogen atoms of the pyrimidine rings [Hg-N 2.669(8) and 2.677(9) Å], while only the Fe-Hg bond is present for Y = Br and Y = I [69]. In the Group 8 derivatives [M(HgI_2_)(CO)_3_(PPh_2_CH_2_morph)_2_] [M = Fe, Ru; PPh_2_CH_2_morph = *N*-(diphenylphosphinomethyl)morpholine], the M-Hg bonds are 2.665(1) Å [M = Fe] and 2.7075(4) Å [M = Ru]. The shortest Hg-N distances are above 2.7 Å, indicating very weak mercury–nitrogen interactions [70,71]. On the other hand, in the complex [Pt{2,6-(Me_2_NCH_2_)_2_C_6_H_3_}{μ-(*p*-tolyl)NC(H)N(*^i^*Pr)}HgBrCl] (Figure 7b) a formamidinate ligand behaves as bridge between a {Pt(N^C^N)} fragment and HgClBr. The Hg-N bond length is short, 2.156(11) Å, comparable with the Pt-N(formamidinate) one, 2.155(9) Å. Thanks to the Hg-Pt bond, 2.8331(7) Å, the platinum center assumes a pseudo-square-pyramidal geometry [72].

**Figure 8:**
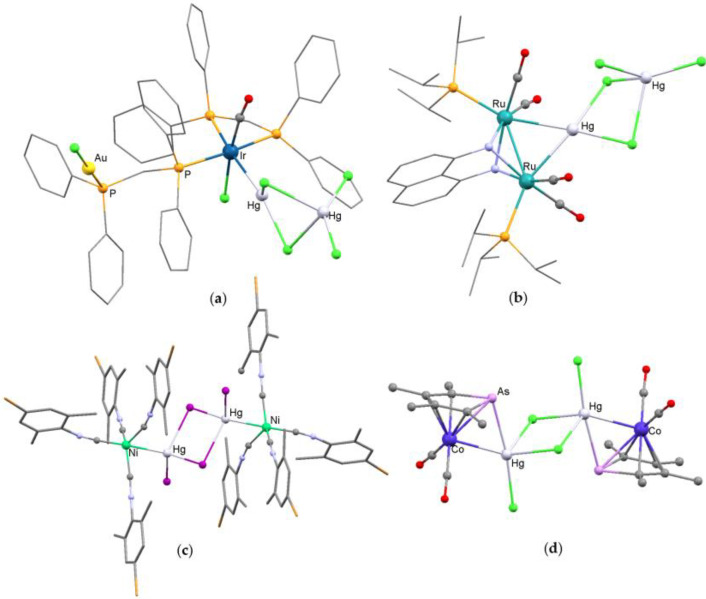
Molecular structures of (**a**) [Ir{Hg(μ-Cl)_2_HgCl_2_}Cl(CO)(dppm){(μ-dppm)AuCl}] [73]; (**b**) [Ru_2_{Hg(μ-Cl)_2_HgCl_2_}(C_10_H_8_N_2_)(CO)_4_(P^i^Pr_3_)_2_] [C_10_H_10_N_2_ = 1,8-diaminonaphthalene] [75]; (**c**) [Ni(CNAr)_4_{HgI(μ-I)_2_HgI}Ni(CNAr)_4_] [Ar = 4-Br-2,6-Me_2_C_6_H_2_] [77]; (**d**) [{HgCl(μ-Cl)_2_HgCl}{Co(η^5^-C_4_Me_4_As)(CO)_2_}_2_] [80]. Color map: Hg, light grey; Au, yellow; Ir, dark blue; Ru, bluish green; Ni, green; Co, blue; I, purple; Br, dark orange; Cl, light green; As, violet; P, orange; O, red; C, grey. Hydrogen atoms omitted.

The formation of a M_m_-HgY_2_ bond is not the only potential outcome from the reaction between a mercury(II) halide and a transition metal precursor or from the insertion of mercury in the M-Y bond. One of the most common possibilities is the presence in the molecular structure of fragments having general formula {Hg_2_Y_2_(μ-Y)_2_}, which can act as formal terminal ligands [73] or behave as bridges between two transition metal centers, these last connected [74,75] or not [76,77,78] by a M-M bond. The structures of [Ir{Hg(μ-Cl)_2_HgCl_2_}Cl(CO)(dppm){(μ-dppm)AuCl}] [Hg-Ir 2.618(3) Å], [Ru_2_{Hg(μ-Cl)_2_HgCl_2_}(C_10_H_8_N_2_)(CO)_4_(P^i^Pr_3_)_2_] [C_10_H_10_N_2_ = 1,8-diaminonaphthalene; Hg-Ru 2.758(1) and 2.775(2) Å; Ru-Ru 2.827(2) Å] and [Ni(CNAr)_4_{HgI(μ-I)_2_HgI}Ni(CNAr)_4_] [Ar = 4-Br-2,6-Me_2_C_6_H_2_; Hg-Ni 2.619(3) Å] are shown as examples in Figure 8a–c. As for HgY_2_, the interaction of {HgY(μ-Y)_2_YHg} with the transition metal fragment can be supported by the coordination of suitable donor groups present in the ancillary ligands, as shown by Zhang and co-workers using 2-pyridylphosphines as bridging ligands between iron carbonyls and mercury [79]. For instance, in the structure of [Fe{Hg(μ-Cl)_2_HgCl_2_}(CO)_4_(μ-PPh_2_py)] one of the mercury centers interacts both with iron [Hg-Fe 2.570(2) Å] and with nitrogen [Hg-N 2.483(11) Å]. Hill and Kirk recently provided another example with the arsolyl-complex [{HgCl(μ-Cl)_2_HgCl}{Co(η^5^-C_4_Me_4_As)(CO)_2_}_2_]. Two isomers of the compound exist, which differ in the mutual *syn* or *anti* positions of the {Co(η^5^-C_4_Me_4_As)(CO)_2_} fragments with respect to the rhomboidal {HgCl(μ-Cl)_2_HgCl} core. Besides the Hg-Co bonds [2620(1)–2.6702(9) Å], Hg-As interactions are present, with distances comprised between 2.6334(6) and 2.7268(9) Å [80]. One of the isomers is shown in Figure 8d.

**Figure 9:**
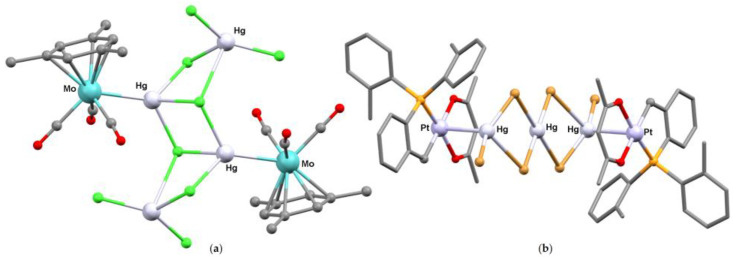
Molecular structures of (**a**) [{Hg_2_(μ_3_-Cl)_2_(μ-Cl)_2_(HgCl_2_)_2_}{Mo(η^6^-C_6_H_3_Me_3_)(CO)_3_}_2_] [81]; (**b**) [{Hg_3_(µ-Br)_4_Br_2_}{Pt(C∧P)(acac)}_2_] [C∧P = CH_2_-C_6_H_4_-P(*o*-tolyl)_2_; acac = 2,4-pentanedionato] [82]. Color map: Hg, light grey; Pt, light violet; Mo, light blue; Br, dark orange; Cl, light green; P, orange; O, red; C, grey. Hydrogen atoms omitted.

**Scheme 1:**
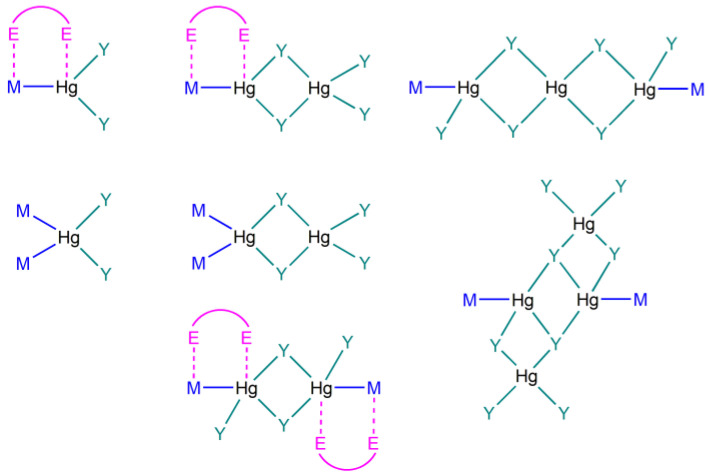
Sketches of the main coordination modes of the {HgY_2_}_n_ fragments (n = 1–4) described in Section 2. Y = halide, E = donor atom.

It is worth noting that the nuclearity of the {HgY_2_}_x_ fragments can be also higher. The compound [{Hg_2_(μ_3_-Cl)_2_(μ-Cl)_2_(HgCl_2_)_2_}{Mo(η^6^-C_6_H_3_Me_3_)(CO)_3_}_2_] (Figure 9a) is composed of two organometallic molybdenum complexes joined by a {HgY_2_}_4_ unit. Two of the four mercury centers form Hg-Mo bonds [2.745(1) Å] [81]. The reaction of [Pt(C∧P)(acac)] [C∧P = CH_2_-C_6_H_4_-P(*o*-tolyl)_2_; acac = 2,4-pentanedionato] with HgBr_2_ afforded the structurally characterized polynuclear species [{Hg_3_(µ-Br)_4_Br_2_}{Pt(C∧P)(acac)}_2_] (Figure 9b), where the terminal mercury centers of the trimercury hexahalide fragment form two unsupported Hg-Pt bonds [2.808(1) Å]. The central mercury atom shows an uncommon square-planar environment [82]. The main coordination modes of the {HgY_2_}_n_ fragments described in this section are sketched in Scheme 1.

## 3. Transition Metal–Mercury Monohalide Derivatives

The apparently simplest cases of transition metal–mercury halide derivatives are species having general formula L_n_M_n_-HgY, where a mercury monohalide formally behaves as a ligand in the coordination sphere of a transition metal center. Intriguing features are the isolobality of the {HgY} fragment with the hydrogen atom and the possibility of intramolecular {YHg···HgY} or intermolecular {YHg···YHg} interactions (*vide infra*).

As for the L_n_M_n_-HgY_2_ derivatives, a common synthetic approach is based on the reaction of HgY_2_ with suitable precursors (Equation (1)); thus, the formation of L_n_M_n_-HgY instead of L_n_M_n_-HgY_2_ can depend upon the experimental conditions.

[L_n_M_n_] + HgY_2_ → [L_n_M_n_-HgY]Y (1)

**Figure 10:**
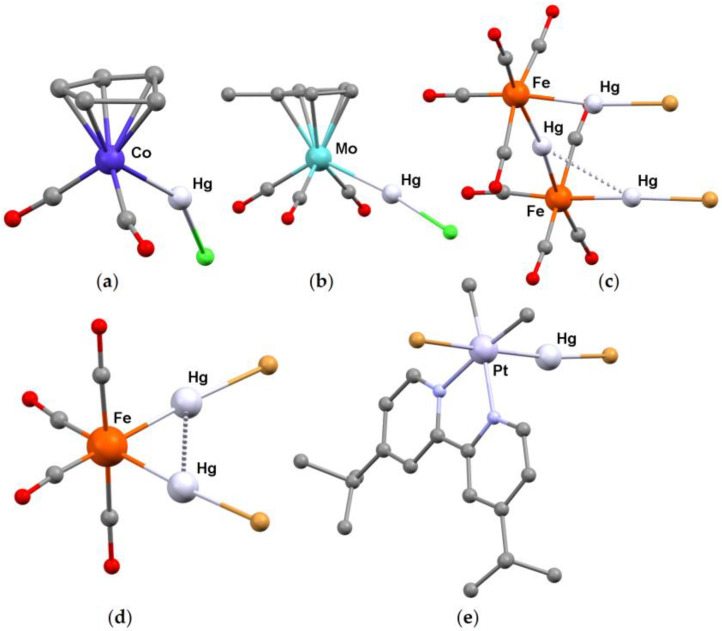
Molecular structures of (**a**) [Co(HgCl)(η^5^-C_5_H_5_)(CO)_2_]^+^ [83]; (**b**) [Mo(HgCl)(η^5^-C_5_H_4_Me)(CO)_3_] [100]; (**c**) [{Fe(HgBr)(CO)_4_}Hg{Fe(HgBr)(CO)_4_}] [113]; (**d**) [Fe(HgBr)_2_(CO)_4_] [115]; (**e**) [Pt(HgBr)BrMe_2_(bpy^Bu2^)] [bpy^Bu2^ =4,4′-di(*tert*-butyl)-2,2′-bipyridine] [95]. Color map: Hg, light grey; Pt, light violet; Mo, light blue; Co, blue; Fe, reddish orange; Br, dark orange; Cl, light green; N, light blue; O, red; C, grey. Hydrogen atoms and intermolecular interactions omitted.

For instance, on increasing the [Co(η^5^-C_5_H_5_)(CO)_2_]:HgCl_2_ ratio, the product isolated was not [Co(HgCl_2_)(η^5^-C_5_H_5_)(CO)_2_] [60], but a 1:3 adduct whose X-ray structure revealed the presence of [Co(HgCl)(η^5^-C_5_H_5_)(CO)_2_]Cl (Figure 10a) and two additional HgCl_2_ molecules [83]. The cation contains a Hg-Co bond [2.504(9) Å] significantly shorter than that found in [Co(HgCl_2_)(η^5^-C_5_H_5_)(CO)_2_] [2.578(4) Å]. A short Hg-Cl bond is present [2.348(16) Å] together with other three Hg---Cl contacts, all above 2.8 Å. The {Co-Hg-Cl} fragment is bent, with an angle of 153.5(5)°. As another example, [Ir(η^5^-C_5_Me_5_)(CO)_2_] reacts with an excess of HgCl_2_ to produce the heterometallic complex [Ir(HgCl)(η^5^-C_5_Me_5_)(CO)_2_][HgCl_3_]. Lowering the amount of HgCl_2_ caused the formation of [Ir(HgCl_2_)(η^5^-C_5_Me_5_)(CO)_2_] as a secondary product [84]. The X-ray crystal structure of [Ir(HgCl)(η^5^-C_5_Me_5_)(CO)_2_][HgCl_3_] shows a nearly linear {Ir-Hg-Cl} group [Hg-Ir 2.5870(11) Å, Hg-Cl 2.354(5) Å, Ir-Hg-Cl 172.12(15)°]. Long Hg···Cl interactions [2.914(6)–3.011(5) Å] connect the cation to the [HgCl_3_]^−^ anion.

Other approaches for the synthesis of {M-Hg-Y} derivatives are based on the cleavage of metal–metal bonds by HgY_2_, using substrates such as L_n_M-Hg-ML_n_ trinuclear complexes, M-SnR_3_ organostannyl species and dinuclear M-M clusters (Equations (2)–(4)) [85,86,87,88,89,90,91]. For instance, species having formulae [M(HgY)(η^5^-C_5_H_5_)(CO)_n_] [M = Mo, n = 3; M = W, n = 3; M = Fe, n = 2; Y = Br, I] and [Co(HgY)(CO)_3_L] [Y = Cl, Br; L = CO or phosphine] were prepared from the corresponding {M-Hg-M} precursors [85,86].

[L_n_M-Hg-ML_n_] + HgY_2_ → 2 [L_n_M-HgY] (2)

[L_n_M-SnR_3_] + HgY_2_ → [L_n_M-HgY] + SnYR_3_ (3)

[L_n_M-ML_n_] + HgY_2_ → [L_n_M-HgY] + [L_n_M-Y] (4)

The oxidative addition of HgY_2_ to a metal center in low oxidation state can also afford {M(HgY)(Y)} complexes (Equation (5)), as shown by the reaction between [Mo(CO)_4_(phen^Me2^)] [phen^Me2^ = 2,9-dimethyl-1,10-phenanthroline] and HgCl_2_, leading to [Mo(HgCl)Cl(CO)_3_(phen^Me2^)] [92]. {Pt^IV^(HgY)Y} derivatives prepared through oxidative addition of HgY_2_ to divalent platinum complexes are further examples of such a synthetic strategy, deeply investigated by Puddephatt and co-workers [93,94,95,96]. Transition metal hydrides can also behave as precursors for the preparation of mercury monohalide derivatives, thanks to the formal exchange between isolobal {HgY} and {H} fragments (Equation (6)). Examples are [Co(HgY)L_4-n_(CO)_n_] [L = phosphite; n = 0–2] complexes obtained from the corresponding hydrides and HgY_2_ [97].

[L_n_M] + HgY_2_ → [L_n_(Y)M-HgY] (5)

[L_n_M-H] + HgY_2_ → [L_n_M-HgY] + HY (6)

Besides [Co(HgCl)(η^5^-C_5_H_5_)(CO)_2_]Cl and [Ir(HgCl)(η^5^-C_5_Me_5_)(CO)_2_][HgCl_3_], several structurally characterized organometallic {M-Hg-Y} derivatives containing a single {HgY} unit are present in the literature, in particular for Group 6 transition metals. In [Mo(HgCl)(η^5^-C_5_H_4_R)(CO)_3_] [R = H, Me], the molybdenum center is seven-coordinated, and the Hg-Mo distance is unaffected by the substitution of the cyclopentadienyl ring [2.683(1) Å for R = H, 2.680(2) Å for R = Me]. The Hg-Cl bonds are comprised between 2.442(3) Å [R = H] and 2.398(5) Å [R = Me]. The {Mo-Hg-Cl} fragment is more bent in the cyclopentadienyl derivative [160.02(9)°] with respect to the methylcyclopentadienyl complex [172.0(1)°]. Both the compounds show contacts between {HgCl} fragments belonging to neighboring molecules, with Hg···Cl distances slightly above 3.0 Å [98,99,100]. [Mo(HgCl)(η^5^-C_5_H_4_Me)(CO)_3_] is depicted as an example in Figure 10b. Further analogous compounds with substituted cyclopentadienyl rings were synthesized and characterized, and the interest was focused on the ^95^Mo and ^199^Hg chemical shift values [101]. The cyclopentadienyl rings can be formally replaced by isolobal ligands, such as boratabenzenes. The structure of the complex [Mo(HgCl)(η^5^-3,5-Me_2_C_5_H_3_BN*^i^*Pr_2_)(CO)_3_}] is comparable with that of [Mo(HgCl)(η^5^-C_5_H_4_Me)(CO)_3_] [102]. The Mo-Hg bond appears scarcely affected also by the replacement of one of the carbonyl ligands with a trivalent Group 15 ligand. For instance, the Hg-Mo distance in [Mo(HgI)(η^5^-C_5_H_4_Me)(CO)_2_(AsPhMe_2_)] is 2.685(3) Å, in line with the previous examples. The Hg-I bond length is 2.720(3) Å and the Mo-Hg-I angle is 167.40(8)°. The intramolecular Hg···I distances are long, 3.561(3) Å [103]. The related [W(HgCl)(η^5^-C_5_H_5_)(CO)_2_(PPh_3_)] complex, obtained by reacting [Hg{W(η^5^-C_5_H_5_)(CO)_3_}_2_] with PPh_3_ in the presence of chlorinated solvents, shows Hg-W and Hg-Cl bond lengths respectively equal to 2.667(1) and 2.382(4) Å and a W-Hg-Cl angle of 173.8(1)° [104]. For what concerns non-cyclopentadienylic Group 6 derivatives, the structures of [Mo(HgCl)Cl(CO)_3_(N-N)] [N-N = 2,2′-bipyridine, 2,9-dimethyl-1,10-phenanthroline] complexes were reported. The Hg-Mo bond lengths are between 2.700(7) and 2.724(2) Å, slightly longer than in the previously described molybdenum-mercury compounds [92,105].

No {M-Hg-Y} complex of Groups 3 and 4 elements is present in the literature. For what concerns Group 5 derivatives, the proposed general formula for the unique compounds synthesized is [Nb(HgY)_2_H(η^5^-C_5_H_5_)_2_]·xHgY_2_ [Y = Cl, Br, I; x = 0.5–1], but the characterization data are not supported by X-ray structure diffraction [106]. Crystal structures of dinuclear {M-Hg-Y} compounds belonging to Group 7 are also absent. Despite the fact that complexes of the type [M(HgY)(CO)_5_] [M = Mn, Re] were prepared from the cleavage of M-Sn or M-Ln^II^ [Ln^II^ = divalent lanthanide] bonds by HgY_2_, the characterization data are limited to elemental analyses and IR spectra [90,107]. Only spectroscopic data are available also for the carbonyl complexes [Mn(HgBr)(η^5^-C_5_H_4_Me)(SiPh_2_Me)(CO)_2_] [108], [Re(HgCl)(η^5^-C_4_H_4_BPh)(CO)_3_] [109] and [Re(HgY)_2_(η^5^-C_5_H_5_)(CO)_2_] [Y = Br, I] [110]. As for [Nb(HgY)_2_H(η^5^-C_5_H_5_)_2_]·xHgY_2_, the last species is described as containing two {HgY} fragments interacting with the same rhenium center. The only technetium derivative reported is [Tc(HgBr)(NAr)_3_] [Ar = 2,6-diisopropylphenyl] [111], but also in this case the X-ray structure is absent.

An example of a structurally characterized {M-Hg-Y} Group 8 derivative with unsupported Hg-M interaction is *mer*-[Fe(HgBr)(SiMePh_2_)(PMe_3_)(CO)_3_]. The Hg-Fe and Hg-Br bond lengths are 2.515(3) and 2.535(3) Å, respectively, and the Fe-Hg-Br angle is 161.0(1)°. The compound is a dimer at the solid state thanks to a second Hg···Br interaction, equal to 3.063(1) Å [112]. The compounds [{Fe(HgY)(CO)_4_}Hg{Fe(HgY)(CO)_4_}] [Y = Cl, Br] [113] allow the comparison between the Hg-M bonds involving {HgY} and {μ-Hg} fragments in the same molecule. The interactions of the iron centers with the bridging mercury atom are longer [2.562(2)–2.570(2) Å for Y = Cl; 2.637(3)–2.638(4) Å for Y = Br] than those with the mercury monohalides, in particular when Y = Br [2.518(2)–2.522(2) Å for Y = Cl; 2.351(3)–2.385(3) Å for Y = Br]. The structure of [{Fe(HgBr)(CO)_4_}Hg{Fe(HgBr)(CO)_4_}] is depicted in Figure 10c. Besides the Hg-Y bonds, in both the structures additional Hg···Y long interactions are present, above 3.1 Å. Another noticeable example of iron–mercury complex is [Fe(HgCl)_2_(CO)_4_], discovered in 1928 [114], where two {HgY} fragments interact with the same transition metal center. The structure of the related [Fe(HgBr)_2_(CO)_4_] complex (Figure 10d) revealed the presence of mercurophilic interaction between the *cis*-{HgBr} fragments, with Hg···Hg distance around 3.0–3.1 Å [115]. One or two carbonyl ligands of [Fe(HgY)_2_(CO)_4_] can be replaced by N-donor ligands [53]. The synthesis and reactivity of other {Fe-Hg-Y} complexes are reported in the literature, such as [Fe(HgY)(NO)(CO)_3_] [116] and [Fe(HgY)(η^5^-C_5_H_5_)(CO)_2_] [90,117,118], but unfortunately data from single-crystal X-ray diffraction were not collected. Bond lengths and angles are instead available for the osmium derivative [Os(HgCl)(NO)Cl_2_(PPh_3_)_2_], formed by oxidative addition of HgCl_2_ to [OsCl(CO)(NO)(PPh_3_)_2_] and loss of the carbonyl ligand. The transition metal center is six-coordinated and the Hg-Os distance is 2.577(6) Å. The {Os-Hg-Cl} fragment is almost linear, being the angle 177(1)° [119].

Besides [Co(HgCl)(η^5^-C_5_H_5_)(CO)_2_]Cl [83] and [Ir(HgCl)(η^5^-C_5_Me_5_)(CO)_2_][HgCl_3_] [84], examples of structurally characterized Group 9 {M-Hg-Y} complexes are [Co(HgY){P(OPh)_3_}_4_] [Y = Cl, Br] derivatives [120]. The Hg-Co distance is almost unaffected by the choice of the halide, falling in the 2.481(2)–2.485(2) Å range. The Co-Hg-Y angles are also strictly comparable, being 163.1(1) and 163.8(1)°. Unfortunately, the bond lengths and angles are not available for the related tetracarbonyl complexes [121]. In the case of iridium, crystal data were reported for [Ir(HgCl)Cl_2_(CO)(PPh_3_)_2_], dimer at the solid state [Hg-Ir 2.570(1) Å, [Hg-Cl 2.366(5) Å, Hg···Cl 3.148(5) Å, Ir-Hg-Cl 172.2(1)°], and for the isomorphous bromo-derivative [Ir(HgBr)ClBr(CO)(PPh_3_)_2_], both derived from the oxidative addition of HgY_2_ to Vaska’s complex *trans*-[IrCl(CO)(PPh_3_)_2_] [122]. Other compounds have the general formula [Ir(HgCl){CCCW(Tp^R2^)(CO)_2_}_2_(CO)(PPh_3_)_2_] [Tp^R2^ = tris(pyrazol-1-yl)borate, tris(3,5-dimethylpyrazol-1-yl)borate]. The Ir-Hg distances are between 2.5905(16) and 2.615(3) Å, while the {Ir-Hg-Cl} fragments are almost linear [123]. A recent example of structurally characterized Group 9–HgCl complex is [Ir(HgCl)(C^N^C)(COD)] [H_2_C^N^C = 2,6-bis(4-*tert*-butylphenyl)pyridine] [16]. The Hg-Ir distance is 2.5705(3) Å and the Ir-Hg-Cl angle is 171.64(4)°.

X-ray data for binuclear {M-Hg-Y} compounds with the transition metal center belonging to Group 10 and unsupported M-Hg bonds are available for M = Pd and M = Pt. As described before, {Pt^IV^(HgY)Y} complexes can be obtained from the oxidative addition of HgY_2_ to suitable Pt(II) precursors, such as square-planar species with two C-donors and a bidentate N-donor in the coordination sphere [93,94,95,96]. The Hg-Pt distance is usually slightly above 2.5 Å and dimerization at the solid state can occur thanks to long Hg···Y interactions. The structure of [Pt(HgBr)BrMe_2_(bpy^Bu2^)] [bpy^Bu2^ =4,4′-di(*tert*-butyl)-2,2′-bipyridine] is shown as an example in Figure 10e. HgCl_2_ also reacts with the trinuclear cluster [Pt_2_Pd(μ-dpmp)_2_{CN(2,6-Me_2_C_6_H_3_)}_2_]^2+^ [dpmp = bis(diphenylphosphinomethyl)phenylphosphine], with the break of the Pt-Pd bond and the formation of [Pt_2_Pd(HgCl)(Cl)(μ-dpmp)_2_{CN(2,6-Me_2_C_6_H_3_)}_2_]^2+^, where the {HgCl} fragment is bonded to palladium [Hg-Pd 2.5830(5) Å], while the other halide interacts with one of the platinum centers. The mercury–palladium bond was described as Hg^I^-Pd^I^, and the additional interaction of mercury with one of the platinum atoms is suggested by the Hg···Pt distance equal to 2.8191(3) Å [124]. On considering another synthetic approach, the reaction of [PtH(PP_3_)]^+^ [PP_3_ = tris(2-diphenylphosphinoethyl)phosphine] with PhHgCl gave [Pt(HgCl)(PP_3_)]^+^, where the {HgCl} fragment occupies one of the apical positions of a trigonal bipyramid surrounding the platinum center. The Hg-Pt bond length is 2.5511(9) Å and the Pt-Hg-Cl angle is 174.63(7)° [125].

**Figure 11:**
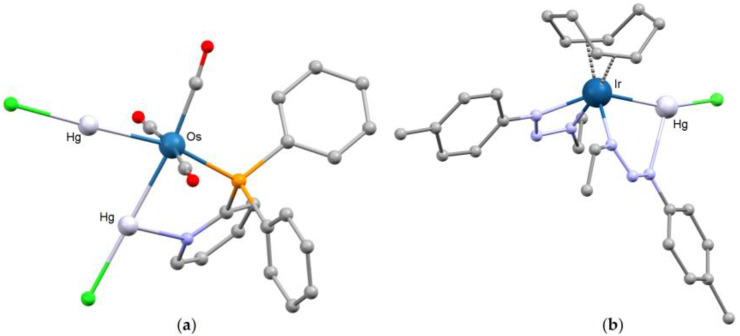
Molecular structures of (**a**) *cis*-[Os(HgCl)_2_(PPh_2_py)(CO)_3_] [PPh_2_py = diphenyl-2-pyridylphosphine] [130]; (**b**) [Ir(HgCl){EtN_3_(4-Me-C_6_H_4_)}_2_(COD)] [COD = 1,5-cyclooctadiene] [132]. Color map: Hg, light grey; Ir, dark blue; Os, blue; Cl, light green; P, orange; N, light blue; O, red; C, grey. Hydrogen atoms omitted.

As previously described for HgY_2_ and {HgY(μ-Y)_2_YHg}, also the interaction of the {HgY} fragment with a transition metal center can be enforced by the presence of donor atoms in the ancillary ligands able to interact with mercury. Functionalized phosphines such as 2-pyridylphosphine, tri(2-furyl)phosphine and related species allowed the preparation of compounds such as [Ru(HgCl)(PPh_2_py)_2_(CO)_3_][HgCl_3_], [Fe(HgI)(CO)_3_(Ph_2_PQu)_2_][HgI_3_] [PhPQu = 2-diphenylphosphino-4-methylquinoline], [M(HgCl)Cl_2_(CO)(PPh_2_py)_2_] [M = Rh, Ir] and [Rh(HgCl)(CO)Cl_2_{P(C_4_H_3_O)_3_}_2_], where Hg···N or Hg···O interactions support the Hg-M bonds [126,127,128,129]. In the *cis*-[M(HgY)_2_(PPh_2_py)(CO)_3_] [M = Ru, Y = Br; M = Os, Y = Cl] derivatives [130], one of the two {HgY} fragments shows an unsupported bond with the transition metal [Hg-Ru 2.602(2) Å; Hg-Os 2.627(1) Å], while the other one also interacts with the nitrogen atom of the pyridine fragment [Hg-Ru 2.628(2) Å, Hg-N 2.772(2) Å; Hg-Os 2.651(1) Å, Hg-N 2.67(1) Å]. Despite this difference, the Hg-Y bonds and M-Hg-Y angles in the same molecule are very similar [Hg-Br 2.540(3) and 2.538(4) Å; Hg-Cl 2.392(6) and 2.400(5) Å; Ru-Hg-Br 165.4(1) and 169.5(1)°; Os-Hg-Cl 177.5(1) and 176.4(1)°]. The structure of the osmium derivative is shown in Figure 11a.

The possibility of quite strong Hg-N interactions with the {M-Hg-Y} fragment is highlighted by the crystal structure of [Fe{HgCl(py)}_2_(CO)_4_], where the Hg center forms bonds with Fe [2.552(8) Å], Cl [2.61(1) Å] and the nitrogen atom of pyridine [2.51(6) Å]. The description of the compound at the solid state must, however, also account for a further intermolecular Hg···Cl interaction equal to 2.771(1) Å [131]. Another example is the triazenido-complex [Ir(HgCl){EtN_3_(4-Me-C_6_H_4_)}_2_(COD)] [Hg-Ir 2.618(1) Å], where one of the two triazenido-ligands bridges the iridium and mercury centers [Hg-N 2.42(1) Å] (Figure 11b). Mercury also forms an intramolecular [2.41(1) Å] and an intermolecular [3.08(1) Å] Hg-Cl bond [132].

**Figure 12:**
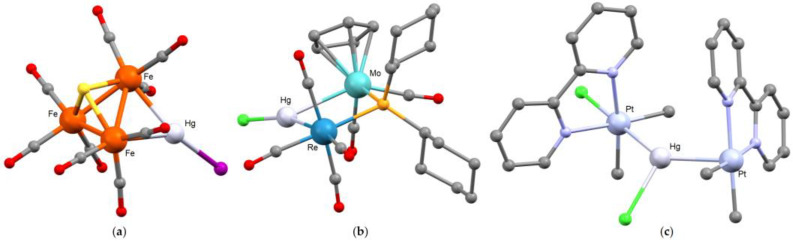
Molecular structures of (**a**) [Fe_3_(HgI)S(CO)_9_]^−^ [133]; (**b**) [{Re(CO)_4_Mo(η^5^-C_5_H_5_)(CO)_2_(HgCl)(μ-PCy_2_)] [18]; (**c**) [{PtMe_2_(phen)}{HgCl}{PtMe_2_Cl(phen)}] [phen = 1,10-phenanthroline] [141]. Color map: Hg, light grey; Pt, light violet; Re, blue; Mo, light blue; I, purple; Cl, light green; S, yellow; P, orange; N, light blue; O, red; C, grey. Hydrogen atoms omitted.

{HgY} can formally behave both as a terminal and bridging ligand. For example, treatment of the trinuclear clusters [Fe_3_E(CO)_9_]^2−^ [E = S, Se] with HgI_2_ afforded the {HgI}-bridged species [Fe_3_(HgI)E(CO)_9_]^−^ (Figure 12a, E = S). The mercury center forms in both the cases two almost identical Hg-Fe bonds [2.6384(8) and 2.6385(7) Å for E = S; 2.605(2) and 2.608(2) Å for E = Se]. The Fe-Hg-Fe angles of the metallacycles are between 64.23(2) and 67.26(6)°, while the Hg-I bonds are between 2.641(1) and 2.6985(4) Å [133,134]. Another Group 8 cluster where {HgY} forms two Hg-M bonds [2.612(3) and 2.862(3) Å] is [Ru_5_C(HgCl)(CO)_14_(μ-Cl)], which is reported as a dimer because of the presence of an additional long intermolecular Hg···Cl interaction [2.961(11) Å] in addition to the Hg-Cl bond [2.412(10) Å]. However, the dissociation of the dimer in solution was proposed [13]. The previously described [Hg{Ru_3_(μ_3_-ampy)(CO)_9_}_2_] cluster [14] reacts with HgBr_2_ to form [Ru_3_(HgBr)(μ_3_-ampy)(CO)_9_], where a {Ru_2_Hg} triangle is present [Hg-Ru 2.735(2) and 2.744(2) Å, Ru-Hg-Ru 63.79(6)°]. Roughly comparable species are the clusters [Os_3_(HgCl)(μ_3_-C_2_Ph_2_)(μ-Cl)(CO)_9_] and [Ru_3_(HgBr)(CO)_9_(C_6_H_9_)], both showing dimerization at the solid state thanks to intermolecular Hg···Y interactions [135,136]. Dimerization at the solid state is not the only possibility when interactions among {HgY} fragments belonging to different molecules occur. An example is provided by the structure of [Os_3_(HgI)(CO)_10_(μ-η^1^-Ph)], which is reported as a tetramer. A pseudo-cubic central {Hg_4_I_4_} unit is present in the X-ray structure, where each mercury atom is connected to three iodides [2.9112(8), 2.9645(8) and 3.524(1) Å]. The I-Hg-I angles are 87.29(2) and 91.11(2)°. Moreover, each mercury center forms two Hg-Os bonds [2.7930(6) and 2.7978(7) Å] [137].

Examples of clusters with bridging {HgY} based on metal centers belonging to other Groups are [Rh_2_(HgCl)(μ-H)(CO)_2_{μ-(PhO)_2_PN(Et)P(OPh)_2_}_2_] [Hg-Rh 2.711(1) and 2.778(1) Å] [138], [Re_2_(HgI)(CO)_8_(μ-η^1^-C_6_H_5_)] [Hg-Re 2.7843(8) and 2.8051(7) Å] [139], [{Re_2_(HgCl)(CO)_8_(μ-PCy_2_)] [Hg-Re 2.777(1) and 2.784(1) Å] and [{Re(CO)_4_Mo(η^5^-C_5_H_5_)(CO)_2_(HgCl)(μ-PCy_2_)] [18]. In the last compound (Figure 12b), there are Hg-Re [2.707(1) Å] and Hg-Mo [2.896(1) Å] bonds and the Re-Hg-Mo angle is 73.6(1)°. Intermolecular Hg···Y interactions at the solid state are common for these species.

Despite the presence of two identical transition metals, the structure of [RhCl(PPh_3_)(CO)(HgCl)(μ-pz)_2_Rh(CO)(PPh_3_)] [pz = pyrazolate] is described as a {HgCl} fragment bridging Rh(I) and Rh(III) centers, with Hg-Rh^I^ and Hg-Rh^III^ distances respectively equal to 2.804(3) and 2.586(2) Å [140]. The nature of the M-Hg bonds is a matter of discussion also in trinuclear platinum-mercury derivatives. The compound [HgBr{PtMe_2_Cl(bpy^Bu2^)}{PtMe_2_(bpy^Bu2^)}] [95,96] shows a covalent Pt^IV^-HgBr bond [2.5767(7) Å], but the same {HgBr} fragment is also involved in a second Pt→Hg donor-acceptor interaction with a Pt(II) center [2.6973(4) Å]. Yamaguchi and Yoshiya isolated two coordination isomers of a trinuclear heterometallic derivative having general formula [HgPt_2_(CH_3_)_2_Cl_4_(phen)]_2_] [phen = 1,10-phenanthroline]. In the first isomer, [Hg{PtMe_2_Cl(phen)}_2_], the mercury atom forms two covalent Hg-Pt bonds [2.5459(8) and 2.5483(7) Å] and the two platinum centers are, including the bonds with mercury, six-coordinated. In the other isomer, [{PtMe_2_(phen)}{HgCl}{PtMe_2_Cl(phen)}] (Figure 12c), {HgCl} behaves as bridge between two platinum fragments. A covalent bond is present with the six-coordinated platinum [Hg-Pt 2.5744(6) Å], while a longer dative Pt→Hg bond, 2.6635(7) Å, joins a square-planar platinum fragment and {HgCl}, with the formation of a square-pyramidal geometry [141].

**Figure 13:**
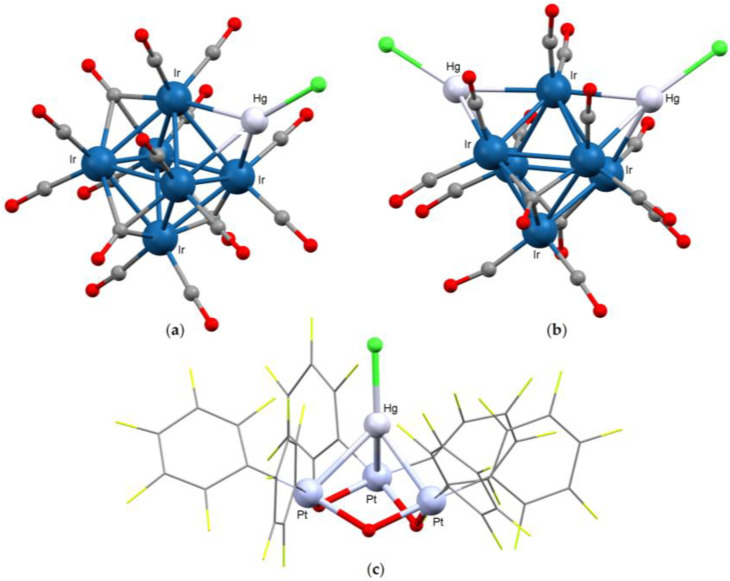
Molecular structures of (**a**) [Ir_6_(HgCl)(CO)_15_]^−^ [142]; (**b**) [Ir_6_(CO)_14_(HgCl)_2_]^2−^ [143]; (**c**) [Pt_3_(HgCl)(μ-OH)_3_(C_6_F_5_)_6_]^2−^ [153]. Color map: Hg, light grey; Pt, light violet; Ir, dark blue; Cl, light green; F, greenish yellow; O, red; C, grey. Hydrogen atoms omitted.

**Scheme 2:**
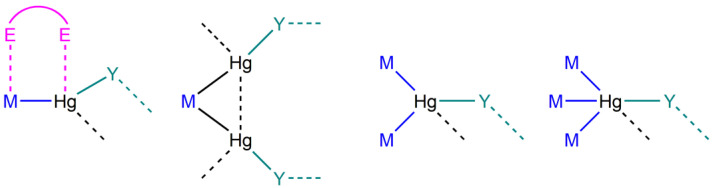
Sketches of the main coordination modes of {HgY} described in Section 3. Y = halide, E = donor atom.

{HgY} can also interact with three metal centers of the same cluster, as observable in the structures of [Ir_6_(HgCl)(CO)_15_]^−^ and [Ir_6_(CO)_14_(HgCl)_2_]^2−^ (Figure 13a,b), where one or two faces of the {Ir_6_} octahedra are capped by {HgCl} fragments, with the formation of three Hg-Ir bonds in the 2.768(2)–2.808(2) Å range [142,143]. Capping {HgY} fragments are present also in the structure of [Pt_6_(HgI)_2_(μ-CO)_6_(μ-dppm)_3_], with Hg-Pt distances varying from 2.770(2) to 2.867(1) Å [144]. A yet more complex situation was observed in the cluster [{Hg_2_Br_2_}{Pt_3_(HgBr)(μ-CO)_3_(PPhCy_2_)_3_}_2_] [145], where four {HgBr} fragments are present. Two of them compose a central {Hg_2_Br_2_} square-planar arrangement of two mercury atoms and two bridging bromine atoms, with Hg-Br bond lengths around 2.7 Å. The other two {HgBr} behave as capping ligands toward the two {Pt_3_} units. Each mercury atom forms three Hg-Pt bonds [Hg-Pt 2.853(1) Å]. The three M-Hg bonds can have quite different lengths, as in the clusters [Os_10_C(HgBr)(CO)_24_]^−^ [Hg-Os bonds between 2.730(2) and 2.924(2) Å] [146], [Pd_4_(HgBr)_2_(CO)_4_(PEt_3_)_4_] [Hg-Pd bonds between 2.704(1) and 2.993(1) Å] [147,148], [Pt_4_(HgBr)_2_(μ-CO)_4_(PPh_3_)_4_] [Hg-Pt bonds between 2.736(1) and 3.113(1) Å] [149] and [Pt_4_(HgI)_2_(μ-CO)_4_(PMe_2_Ph)_4_] [Hg-Pt between 2.716(3) and 3.163(3) Å] [150]. The transition metals can be also different, as occurs in the cluster [Pt_3_Ru_6_(HgI)(µ_3_-H)_2_(CO)_21_]^−^, where the mercury center forms two Hg-Ru [2.741(2)–2.774(2) Å] and one Hg-Pt [2.893(1) Å] bonds [151]. The presence of M–M interactions is, however, not mandatory. In [Rh_3_(HgCl)(μ-Cl)_3_(dpmppp)L_2_]^+^ [dpmppp = *meso*-1,3-bis[(diphenylphosphinomethyl)phenylphosphino]propane; L = CO, CN(2,6-Me_2_C_6_H_3_)] the three rhodium centers are connected by a tetradentate phosphine, three bridging chloro-ligands and a {HgCl} fragment forming three Hg-Rh bonds in the 2.6408(8)–2.7414(9) Å range [152]. In [Pt_3_(HgCl)(μ-OH)_3_(C_6_F_5_)_6_]^2−^ (Figure 13c), the three platinum atoms are connected by three μ-hydroxo ligands and {HgCl} [Hg-Pt 2.750(1)–2.875(1) Å] [153]. The main coordination modes of {HgY} described in this section are sketched in Scheme 2.

## 4. Computational Investigations on {M-Hg-Y} Derivatives

**Figure 14:**
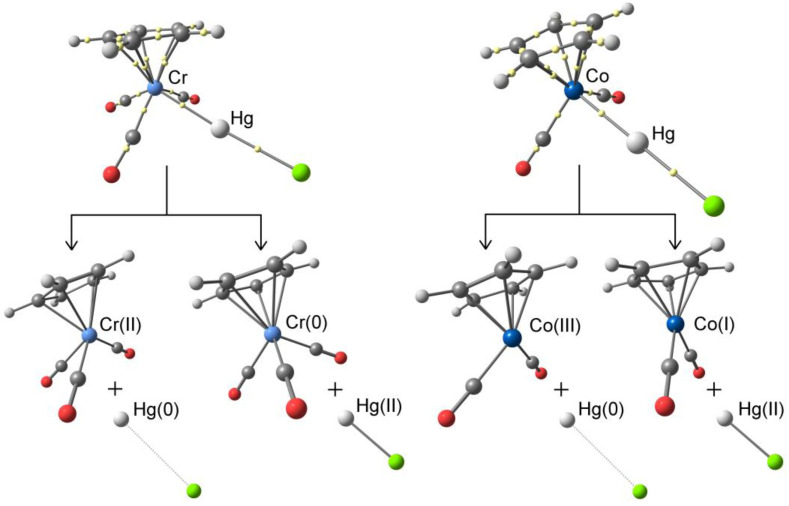
DFT-optimized structures of [Cr(HgCl)(η^5^-C_5_H_5_)(CO)_3_] and [Co(HgCl)(η^5^-C_5_H_5_)(CO)_2_]^+^ and products of the heterolytic Hg-M dissociation reactions [C-PCM/r^2^SCAN-3c calculations]. Color map: Hg, light grey; Co, blue; Cr, light blue; Cl, light green; O, red; C, grey; H, white. (3, −1) BCPs represented as small light yellow spheres. Selected computed bond lengths (Å) and angles (°): [Cr(HgCl)(η^5^-C_5_H_5_)(CO)_3_], Hg-Cr 2.611, Hg-Cl 2.431, Hg-Cr-Cl 175.0; [Co(HgCl)(η^5^-C_5_H_5_)(CO)_2_]^+^, Hg-Co 2.495, Co-Cl 2.378, Hg-Co-Cl 179.6. Computed values at Hg-M (3, −1) BCPs [a.u.]: M = Cr, ρ 0.054, V −0.048, E −0.015, $\nabla^{2}$ ρ 0.074; M = Co, ρ 0.065, V −0.059, E −0.017, $\nabla^{2}$ρ 0.102.

As revealed by the previous examples, the formal oxidation states in transition metal–mercury monohalide derivatives are sometimes ambiguous; thus, the {HgY} fragment can be considered as a Lewis acid or base on varying the transition metal fragment. [M(HgY)(η^5^-C_5_H_5_)(CO)_3_] [M = Cr, Mo, W; Y = Cl, Br, I] and [M(HgY)(η^5^-C_5_H_5_)(CO)_2_]^+^ [M = Cr, Mo, W; Y = Cl, Br, I] were selected as model compounds to investigate the M-HgY bonds from a computational point of view. The intermolecular interactions were omitted. Cartesian coordinates of all the optimized geometries from density functional theory (DFT) calculations are provided as Supplementary Materials, together with computed Hg-M and Hg-Y bond lengths and M-Hg-Y angles (Table S1). The stationary points obtained for [Cr(HgCl)(η^5^-C_5_H_5_)(CO)_3_] and [Co(HgCl)(η^5^-C_5_H_5_)(CO)_2_]^+^ are depicted as examples in Figure 14. The M-Hg bond lengths show a slight increase (0.02 Å or less) moving from Y = Cl to Y = I. The M-Hg-Y angles are between 175.0 and 177.5° for Group 6 derivatives and between 177.3 and 179.7° for Group 9 complexes. The lower linearity of the {M-Hg-Y} fragments experimentally observed in a number of cases appears to be attributable to intermolecular interactions such as Hg···Y contacts.

The Gibbs energy variation for the heterolysis reactions [M(HgY)(η^5^-C_5_H_5_)(CO)_x_]^n+^ → [M(η^5^-C_5_H_5_)(CO)_x_]^(n+1)+^ + [HgY]^−^ and [M(HgY)(η^5^-C_5_H_5_)(CO)_x_]^n+^ → [M(η^5^-C_5_H_5_)(CO)_x_]^(n−1)+^ + [HgY]^+^ [n = 0, x = 3, M = Group 6; n = 1, x = 2, M = Group 9] were calculated by means of DFT calculations in the presence of acetone as continuous medium (Table 1). The first dissociation affords [HgY]^−^ anions that behave as Lewis bases toward divalent Group 6 and trivalent Group 9 metal centers. Mercury can be considered as Hg(0). On the other hand, the [HgY]^+^ cations generated by the second pathway contain divalent mercury, and they interact with transition metal fragments that behave as Lewis bases thanks to their electron-rich metal centers.

The heterolysis with the lowest Gibbs energy variation should suggest the most reasonable formal oxidation states for mercury and transition metals in the complexes. As observable in Table 1, the Group 6 and Group 9 derivatives here considered have opposite behavior. The dissociation of [HgY]^−^ from [M(HgY)(η^5^-C_5_H_5_)(CO)_3_] [M = Cr, Mo, W] is the most favorable path, so the compounds appear better described as M(II) complexes where the coordination sites are occupied by three carbonyls, a cyclopentadienyl ligand and [HgY]^−^. It is worth noting the low influence of the nature of both M and Y on the Δ*G* values, comprised between 51.1 and 55.4 kcal mol^−1^. The scarce energy variations on changing Y are in part attributable to the weak interactions between mercury and halides in [HgY]^−^. On the contrary, the heterolytic dissociations of the Hg-M bonds in [M(HgY)(η^5^-C_5_H_5_)(CO)_2_]^+^ [M = Co, Rh, Ir] should preferentially afford the [HgY]^+^ cations. Therefore, in the Group 9 derivatives, the mercury center is probably best described as Hg(II), behaving as Lewis acid towards electron-rich M(I) complexes. As in the previous cases, the choice of M scarcely affects the Gibbs energy variations. On the other hand, the dissociation requires less energy on increasing the atomic number of the halide, probably because of the increased stability of [HgY]^+^ with softer halides.

The outcome of the charge decomposition analysis on [Cr(HgCl)(η^5^-C_5_H_5_)(CO)_3_], partitioned as [Cr(η^5^-C_5_H_5_)(CO)_3_]^+^ and [HgCl]^−^, was {HgCl}→{Cr(η^5^-C_5_H_5_)(CO)_3_} donation of 0.222 electrons, while the opposite process resulted limited to 0.083 electrons. The same analysis on [Co(HgCl)(η^5^-C_5_H_5_)(CO)_2_]^+^, partitioned as [Co(η^5^-C_5_H_5_)(CO)_2_] and [HgCl]^+^, afforded {Co(η^5^-C_5_H_5_)(CO)_3_}→{HgCl} donation of 0.258 electrons, and only 0.021 electrons resulted back-donated. Roughly comparable values were obtained on changing the halide and the metal center, as reported in Table S2.

**Figure 15:**
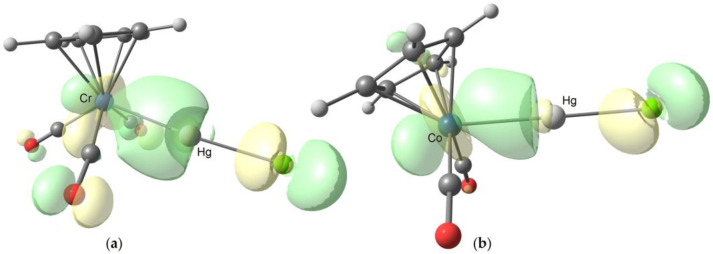
DFT-optimized structures of (**a**) [Cr(HgCl)(η^5^-C_5_H_5_)(CO)_3_] and (**b**) [Co(HgCl)(η^5^-C_5_H_5_)(CO)_2_]^+^ with plots of the occupied molecular orbitals mainly responsible for the Hg-M σ-overlaps. Color map: Hg, light grey; Co, blue; Cr, light blue; Cl, light green; O, red; C, grey; H, white. Surfaces in yellow and green tones, isovalue = 0.04 a.u.

The Atoms-in-Molecules (AIM) analysis on [Cr(HgCl)(η^5^-C_5_H_5_)(CO)_3_] and [Co(HgCl)(η^5^-C_5_H_5_)(CO)_2_]^+^ revealed the presence of Hg-M (3, −1) bond critical points (BCPs) characterized by quite high values of electron density (ρ) and absolute values of potential energy density (V). The energy density (E) values are negative and the Laplacian of electron density ($\nabla^{2}$ρ) is positive, in line with Bianchi’s definition of metal–metal bond [154]. Selected data for the two compounds are collected in the caption of Figure 14. The occupied molecular orbitals mainly responsible for the Hg-M σ-overlaps are shown in Figure 15.

The AIM data for the other compounds, summarized in Table S3, indicate scarce influence of the nature of the halide on the ρ and V values at Hg-M (3, −1) BCP. For what concerns the Hg-Cl (3, −1) BCPs, the ρ and V values highlight a stronger bond in the cobalt derivative [ρ = 0.084 a.u., V = −0.094 a.u.) respect to the chromium complex [ρ = 0.075 a.u., V = −0.082 a.u.]. Such a result appears in line with the greater Lewis acid behavior proposed for the mercury center in [Co(HgCl)(η^5^-C_5_H_5_)(CO)_2_]^+^ respect to [Cr(HgCl)(η^5^-C_5_H_5_)(CO)_3_]. The AIM data at Hg-Y (3, −1) BCP remain roughly constant by replacing the metal center with heavier congeners, as observable in Table S3. The different strength of the Hg-Cl bonds was confirmed by the computed Mayer bond orders [155], equal to 0.670 in [Co(HgCl)(η^5^-C_5_H_5_)(CO)_2_]^+^ and 0.580 in [Cr(HgCl)(η^5^-C_5_H_5_)(CO)_3_]. Despite all the differences, the Hg-M Mayer bond order values in [Cr(HgCl)(η^5^-C_5_H_5_)(CO)_3_] and [Co(HgCl)(η^5^-C_5_H_5_)(CO)_2_]^+^ are strictly comparable, respectively equal to 0.650 and 0.646.

## 5. Computational Methods

The geometry optimizations were carried out with the r^2^SCAN-3c method [156], based on the *meta*-GGA r^2^SCAN functional [157] combined with a tailor-made triple-ζ Gaussian atomic orbital basis set, with relativistic effective core potentials for mercury and the heavier atoms [158,159]. The method also includes refitted D4 and geometrical counter-poise corrections for London dispersion and basis set superposition error [160,161]. The C-PCM implicit solvation model was added, considering acetone as continuous medium [162]. IR simulations were carried out using the harmonic approximation, from which zero-point vibrational energies and thermal corrections (T = 298.15 K) were obtained. Calculations were carried out using ORCA version 5.0.3 [163,164] and the output files were analyzed with Multiwfn version 3.8 [165].

## 6. Conclusions

The chemistry of heteropolymetallic transition metal–mercury compounds is complex and fascinating, and the examples provided in this review do not complete the portrait of possibilities offered by the presence of M-Hg bonds. As an example, iron–mercury clusters with high nuclearities and intriguing structures were isolated and characterized by Fenske and co-workers by reacting [Fe(HgY)_2_(CO)_4_] derivatives with phosphines and related species [166].

The present review is focused on mercury monohalides, whose modes of interaction with transition metal fragments are qualitatively comparable with those of the hydrogen atom thanks to the isolobal analogy. The {HgY} fragment, however, offers peculiar possibilities, such as the displacement of the halide and the presence of intra- and intermolecular mercurophilic and mercury–halide interactions. Mercury is also of interest because of the high atomic number and the consequent relativistic effects induced in transition metal compounds. Despite the well-known toxicity of mercury, which limits the use of its derivatives in synthetic chemistry, mercury monohalides can be considered as unusual ligands able to promote uncommon chemical and physical features in organometallic compounds, of potential interest in the fields of catalysis and functional materials.

## Figures and Tables

**Table 1:** Computed Gibbs energy variations [kcal mol^−1^; C-PCM/r^2^SCAN-3c calculations] for the heterolysis of the M-Hg bonds in selected complexes. C-PCM/r^2^SCAN-3c, acetone as continuous medium.

Complex	[M(HgY)L_n_]^n+^ → [ML_n_]^(n−1)+^ + [HgY]^+^	[M(HgY)L_n_]^n+^ → [ML_n_]^(n+1)+^ + [HgY]^−^
[Cr(HgCl)(η^5^-C_5_H_5_)(CO)_3_]	76.8	52.5
[Cr(HgBr)(η^5^-C_5_H_5_)(CO)_3_]	73.2	51.1
[Cr(HgI)(η^5^-C_5_H_5_)(CO)_3_]	67.2	51.1
[Mo(HgCl)(η^5^-C_5_H_5_)(CO)_3_]	78.6	53.1
[Mo(HgBr)(η^5^-C_5_H_5_)(CO)_3_]	74.8	51.6
[Mo(HgI)(η^5^-C_5_H_5_)(CO)_3_]	68.6	51.3
[W(HgCl)(η^5^-C_5_H_5_)(CO)_3_]	80.1	55.4
[W(HgBr)(η^5^-C_5_H_5_)(CO)_3_]	76.2	53.7
[W(HgI)(η^5^-C_5_H_5_)(CO)_3_]	69.9	53.4
[Co(HgCl)(η^5^-C_5_H_5_)(CO)_2_]^+^	51.1	87.1
[Co(HgBr)(η^5^-C_5_H_5_)(CO)_2_]^+^	47.8	85.9
[Co(HgI)(η^5^-C_5_H_5_)(CO)_2_]^+^	42.7	86.7
[Rh(HgCl)(η^5^-C_5_H_5_)(CO)_2_]^+^	51.9	81.2
[Rh(HgBr)(η^5^-C_5_H_5_)(CO)_2_]^+^	48.5	80.1
[Rh(HgI)(η^5^-C_5_H_5_)(CO)_2_]^+^	43.3	80.8
[Ir(HgCl)(η^5^-C_5_H_5_)(CO)_2_]^+^	55.9	87.6
[Ir(HgBr)(η^5^-C_5_H_5_)(CO)_2_]^+^	52.3	86.2
[Ir(HgI)(η^5^-C_5_H_5_)(CO)_2_]^+^	46.8	86.7

## Data Availability

Data are contained within the article and Supplementary Materials.

## Supplementary Materials

The following supporting information can be downloaded at: https://www.mdpi.com/article/10.3390/molecules30010145/s1, Table S1: Selected computed bond lengths and angles for [M(HgY)(η^5^-C_5_H_5_)(CO)_3_] [M = Cr, Mo, W; Y = Cl, Br, I] and [M(HgY)(η^5^-C_5_H_5_)(CO)_2_]^+^ [M = Cr, Mo, W; Y = Cl, Br, I]; Table S2: Output of the charge decomposition analysis on [M(HgY)(η^5^-C_5_H_5_)(CO)_3_] [M = Cr, Mo, W; Y = Cl, Br, I], partitioned as [M(η^5^-C_5_H_5_)(CO)_3_]^+^ and [HgY]^−^, and on [M(HgY)(η^5^-C_5_H_5_)(CO)_2_]^+^ [M = Cr, Mo, W; Y = Cl, Br, I], partitioned as [M(η^5^-C_5_H_5_)(CO)_2]_ and [HgY]^+^; Table S3: AIM data for the Hg-M and Hg-Y (3, −1) BCPs in [M(HgY)(η^5^-C_5_H_5_)(CO)_3_] [M = Cr, Mo, W; Y = Cl, Br, I] and [M(HgY)(η^5^-C_5_H_5_)(CO)_2_]^+^ [M = Cr, Mo, W; Y = Cl, Br, I]; List S1: Cartesian coordinates of the DFT-optimized structures.
