# Supplementary material for: Mercury Monohalides as Ligands in Transition Metal Complexes
Source: Molecules. 2025 Jan 2;30(1):145. doi: 10.3390/molecules30010145 (PMC11721413; doi:10.3390/molecules30010145)
Supplement: Supplementary file 1 [file molecules-30-00145-s001.zip › molecules-3384930-supplementary.pdf]

Mercury Monohalides as Ligands in Transition Metal Complexes

Matteo Busato [1,2], Jesús Castro [3], Domenico Piccolo [1,4] and Marco Bortoluzzi [1,5]*

[1] Dipartimento di Chimica, Sapienza Università di Roma, P.le Aldo Moro 5, 00185 Rome, Italy; matteo.busato@uniroma1.it (M.B.); domenico.piccolo@unipd.it (D.P.)
[2] Dipartimento di Scienze Molecolari e Nanosistemi, Università Ca' Foscari Venezia, 30172 Mestre, Italy
[3] Departamento de Química Inorgánica, Facultade de Química, Universidade de Vigo, Edificio de Ciencias Experimentais, 36310 Vigo, Galicia, Spain; jesusc@uvigo.gal
[4] Dipartimento di Scienze Chimiche, Università di Padova, Via Marzolo 1, 35131 Padova, Italy
[5] CIRCC (Consorzio Universitario Reattività Chimica e Catalisi), Via Celso Ulpiani 27, 70126 Bari, Italy
* Correspondence: markos@unive.it; Tel.: +39-0412348561

Supplementary Materials

Table S1. Selected computed bond lengths and angles for $[M(HgY)(\eta^5\text{-}C_5H_5)(CO)_3]$ [M = Cr, Mo, W; Y = Cl, Br, I] and $[M(HgY)(\eta^5\text{-}C_5H_5)(CO)_2]^+$ [M = Cr, Mo, W; Y = Cl, Br, I]. C-PCM/r^{2}SCAN-3c calculations.

Complex	Hg-M (Å)	Hg-Y (Å)	M-Hg-Y (°)
$[Cr(HgCl)(\eta^5\text{-}C_5H_5)(CO)_3]$	2.611	2.431	175.0
$[Cr(HgBr)(\eta^5\text{-}C_5H_5)(CO)_3]$	2.619	2.556	175.0
$[Cr(HgI)(\eta^5\text{-}C_5H_5)(CO)_3]$	2.630	2.727	176.9
$[Mo(HgCl)(\eta^5\text{-}C_5H_5)(CO)_3]$	2.721	2.438	175.3
$[Mo(HgBr)(\eta^5\text{-}C_5H_5)(CO)_3]$	2.727	2.562	175.6
$[Mo(HgI)(\eta^5\text{-}C_5H_5)(CO)_3]$	2.737	2.735	177.2
$[W(HgCl)(\eta^5\text{-}C_5H_5)(CO)_3]$	2.736	2.438	175.6
$[W(HgBr)(\eta^5\text{-}C_5H_5)(CO)_3]$	2.742	2.563	176.0
$[W(HgI)(\eta^5\text{-}C_5H_5)(CO)_3]$	2.752	2.734	177.5
$[Co(HgCl)(\eta^5\text{-}C_5H_5)(CO)_2]^+$	2.495	2.378	179.6
$[Co(HgBr)(\eta^5\text{-}C_5H_5)(CO)_2]^+$	2.502	2.502	179.7
$[Co(HgI)(\eta^5\text{-}C_5H_5)(CO)_2]^+$	2.514	2.673	179.7
$[Rh(HgCl)(\eta^5\text{-}C_5H_5)(CO)_2]^+$	2.607	2.376	179.0
$[Rh(HgBr)(\eta^5\text{-}C_5H_5)(CO)_2]^+$	2.614	2.499	179.5
$[Rh(HgI)(\eta^5\text{-}C_5H_5)(CO)_2]^+$	2.627	2.670	179.6
$[Ir(HgCl)(\eta^5\text{-}C_5H_5)(CO)_2]^+$	2.628	2.372	178.0
$[Ir(HgBr)(\eta^5\text{-}C_5H_5)(CO)_2]^+$	2.635	2.494	179.3
$[Ir(HgI)(\eta^5\text{-}C_5H_5)(CO)_2]^+$	2.648	2.667	177.3

Table S2. Output of the charge decomposition analysis on $[M(HgY)(\eta^5\text{-}C_5H_5)(CO)_3]$ [M = Cr, Mo, W; Y = Cl, Br, I], partitioned as $[M(\eta^5\text{-}C_5H_5)(CO)_3]^+$ and $[HgY]^-$, and on $[M(HgY)(\eta^5\text{-}C_5H_5)(CO)_2]^+$ [M = Cr, Mo, W; Y = Cl, Br, I], partitioned as $[M(\eta^5\text{-}C_5H_5)(CO)_2]$ and $[HgY]^+$. C-PCM/r^{2}SCAN-3c calculations. Values in electrons.

**Complex**	$[HgY]^- \rightarrow [M(\eta^5\text{-}C_5H_5)(CO)_3]^+$	$[HgY]^- \leftarrow [M(\eta^5\text{-}C_5H_5)(CO)_3]^+$
$[Cr(HgCl)(\eta^5\text{-}C_5H_5)(CO)_3]$	0.222	0.083
$[Cr(HgBr)(\eta^5\text{-}C_5H_5)(CO)_3]$	0.226	0.083
$[Cr(HgI)(\eta^5\text{-}C_5H_5)(CO)_3]$	0.218	0.087
$[Mo(HgCl)(\eta^5\text{-}C_5H_5)(CO)_3]$	0.219	0.051
$[Mo(HgBr)(\eta^5\text{-}C_5H_5)(CO)_3]$	0.221	0.051
$[Mo(HgI)(\eta^5\text{-}C_5H_5)(CO)_3]$	0.202	0.052
$[W(HgCl)(\eta^5\text{-}C_5H_5)(CO)_3]$	0.194	0.055
$[W(HgBr)(\eta^5\text{-}C_5H_5)(CO)_3]$	0.209	0.054
$[W(HgI)(\eta^5\text{-}C_5H_5)(CO)_3]$	0.214	0.058
	$[M(\eta^5\text{-}C_5H_5)(CO)_2] \rightarrow [HgY]^+$	$[M(\eta^5\text{-}C_5H_5)(CO)_2] \leftarrow [HgY]^+$
$[Co(HgCl)(\eta^5\text{-}C_5H_5)(CO)_2]^+$	0.258	0.021
$[Co(HgBr)(\eta^5\text{-}C_5H_5)(CO)_2]^+$	0.257	0.026
$[Co(HgI)(\eta^5\text{-}C_5H_5)(CO)_2]^+$	0.257	0.028
$[Rh(HgCl)(\eta^5\text{-}C_5H_5)(CO)_2]^+$	0.218	0.000
$[Rh(HgBr)(\eta^5\text{-}C_5H_5)(CO)_2]^+$	0.213	0.000
$[Rh(HgI)(\eta^5\text{-}C_5H_5)(CO)_2]^+$	0.218	0.000
$[Ir(HgCl)(\eta^5\text{-}C_5H_5)(CO)_2]^+$	0.271	0.000
$[Ir(HgBr)(\eta^5\text{-}C_5H_5)(CO)_2]^+$	0.259	0.000
$[Ir(HgI)(\eta^5\text{-}C_5H_5)(CO)_2]^+$	0.257	0.007

Table S3. AIM data for the Hg-M and Hg-Y (3,-1) BCPs in $[M(HgY)(\eta^5\text{-}C_5H_5)(CO)_3]$ [M = Cr, Mo, W; Y = Cl, Br, I] and $[M(HgY)(\eta^5\text{-}C_5H_5)(CO)_2]^+$ [M = Cr, Mo, W; Y = Cl, Br, I]. C-PCM/r^{2}SCAN-3c calculations. ϱ = electron density; V = potential energy density; E = energy density; $\nabla^2 \varrho$ = Laplacian of electron density. Values in atomic units.

**Complex**	ϱ	V	E	$\nabla^2 \varrho$	ϱ	V
		**Hg-M (3,-1)**			**Hg-Y (3,-1)**	
$[Cr(HgCl)(\eta^5\text{-}C_5H_5)(CO)_3]$	0.054	-0.048	-0.015	0.074	0.075	-0.082
$[Cr(HgBr)(\eta^5\text{-}C_5H_5)(CO)_3]$	0.053	-0.048	-0.015	0.073	0.068	-0.064
$[Cr(HgI)(\eta^5\text{-}C_5H_5)(CO)_3]$	0.052	-0.046	-0.014	0.070	0.059	-0.047
$[Mo(HgCl)(\eta^5\text{-}C_5H_5)(CO)_3]$	0.056	-0.046	-0.013	0.080	0.074	-0.081
$[Mo(HgBr)(\eta^5\text{-}C_5H_5)(CO)_3]$	0.055	-0.045	-0.013	0.079	0.067	-0.063
$[Mo(HgI)(\eta^5\text{-}C_5H_5)(CO)_3]$	0.054	-0.044	-0.012	0.077	0.058	-0.046
$[W(HgCl)(\eta^5\text{-}C_5H_5)(CO)_3]$	0.059	-0.048	-0.016	0.066	0.074	-0.081
$[W(HgBr)(\eta^5\text{-}C_5H_5)(CO)_3]$	0.058	-0.048	-0.016	0.066	0.067	-0.064
$[W(HgI)(\eta^5\text{-}C_5H_5)(CO)_3]$	0.057	-0.046	-0.016	0.064	0.058	-0.046
$[Co(HgCl)(\eta^5\text{-}C_5H_5)(CO)_2]^+$	0.065	-0.059	-0.017	0.102	0.084	-0.094
$[Co(HgBr)(\eta^5\text{-}C_5H_5)(CO)_2]^+$	0.064	-0.058	-0.016	0.102	0.075	-0.073
$[Co(HgI)(\eta^5\text{-}C_5H_5)(CO)_2]^+$	0.062	-0.056	-0.015	0.102	0.065	-0.052
$[Rh(HgCl)(\eta^5\text{-}C_5H_5)(CO)_2]^+$	0.066	-0.060	-0.016	0.112	0.084	-0.095
$[Rh(HgBr)(\eta^5\text{-}C_5H_5)(CO)_2]^+$	0.065	-0.059	-0.016	0.112	0.076	-0.074
$[Rh(HgI)(\eta^5\text{-}C_5H_5)(CO)_2]^+$	0.063	-0.058	-0.015	0.110	0.065	-0.053
$[Ir(HgCl)(\eta^5\text{-}C_5H_5)(CO)_2]^+$	0.073	-0.067	-0.021	0.104	0.085	-0.097
$[Ir(HgBr)(\eta^5\text{-}C_5H_5)(CO)_2]^+$	0.072	-0.066	-0.020	0.105	0.077	-0.075
$[Ir(HgI)(\eta^5\text{-}C_5H_5)(CO)_2]^+$	0.070	-0.064	-0.019	0.105	0.066	-0.053

List S1. Cartesian coordinates of the DFT-optimized structures [Å].

19

$[Cr(HgCl)(\eta^5\text{-}C_5H_5)(CO)_3]$

Cr	-0.521814000	-1.272299000	-0.000016000
H	-2.358599000	-0.501282000	2.179004000
C	-2.361042000	-0.839469000	1.151325000
C	-2.363897000	-2.190948000	0.713911000
H	-2.411128000	1.076880000	0.000002000
H	-2.365079000	-3.064833000	1.351516000
C	-2.363889000	-2.190943000	-0.713928000
H	-2.365083000	-3.064823000	-1.351540000
C	-2.361030000	-0.839465000	-1.151334000
H	-2.358570000	-0.501270000	-2.179009000
C	-2.372284000	-0.004804000	-0.000001000
C	0.482954000	-1.107065000	1.545636000
O	1.064196000	-1.107580000	2.549136000
C	0.270701000	-2.920998000	-0.000038000
O	0.773104000	-3.964170000	0.000188000
C	0.482876000	-1.106997000	-1.545677000
O	1.064035000	-1.107597000	-2.549228000
Hg	0.537966000	1.114303000	-0.000012000
Cl	1.325362000	3.414702000	0.000067000

19

$[Cr(HgBr)(\eta^5\text{-}C_5H_5)(CO)_3]$

Cr	-0.525151000	-1.280696000	-0.000012000
H	-2.360045000	-0.507425000	2.178980000
C	-2.363469000	-0.845657000	1.151361000
C	-2.368381000	-2.197103000	0.713949000
H	-2.410370000	1.070791000	-0.000013000
H	-2.370430000	-3.071029000	1.351430000
C	-2.368383000	-2.197109000	-0.713944000
H	-2.370438000	-3.071041000	-1.351418000
C	-2.363471000	-0.845669000	-1.151369000
H	-2.360048000	-0.507446000	-2.178991000
C	-2.373150000	-0.010940000	-0.000008000
C	0.480571000	-1.113402000	1.544524000
O	1.062016000	-1.112648000	2.548096000
C	0.265856000	-2.929590000	0.000000000
O	0.767865000	-3.973105000	0.000091000
C	0.480518000	-1.113357000	-1.544567000
O	1.061925000	-1.112618000	-2.548161000
Hg	0.540639000	1.111200000	-0.000008000
Br	1.372724000	3.528188000	0.000062000

19

$[Cr(HgI)(\eta^5\text{-}C_5H_5)(CO)_3]$

Cr	-0.534621000	-1.288724000	0.000007000
H	-2.371130000	-0.522456000	2.179037000
C	-2.374043000	-0.860533000	1.151371000
C	-2.375073000	-2.211887000	0.714006000
H	-2.425574000	1.056032000	-0.000021000
H	-2.373833000	-3.085936000	1.351326000
C	-2.375073000	-2.211903000	-0.713973000
H	-2.373830000	-3.085967000	-1.351273000
C	-2.374044000	-0.860559000	-1.151369000
H	-2.371131000	-0.522505000	-2.179042000
C	-2.385824000	-0.025609000	-0.000008000
C	0.472901000	-1.110313000	1.542004000
O	1.055057000	-1.103409000	2.545687000
C	0.264525000	-2.932904000	0.000008000
O	0.772555000	-3.973872000	-0.000105000
C	0.472888000	-1.110319000	-1.542000000
O	1.055050000	-1.103414000	-2.545679000
Hg	0.534630000	1.113659000	-0.000001000
I	1.505348000	3.661960000	0.000030000

19

[Mo(HgCl)(η^5-C_5H_5)(CO)$_3$]

Mo	-0.465097000	-1.269745000	-0.000200000
H	-2.495265000	-0.497533000	2.181340000
C	-2.486406000	-0.836545000	1.153812000
C	-2.475243000	-2.189088000	0.716606000
H	-2.536441000	1.079890000	-0.000011000
H	-2.491437000	-3.063903000	1.352808000
C	-2.475419000	-2.189052000	-0.716778000
H	-2.491712000	-3.063837000	-1.353019000
C	-2.486668000	-0.836499000	-1.153922000
H	-2.495732000	-0.497444000	-2.181436000
C	-2.500123000	-0.001730000	-0.000036000
C	0.675190000	-1.114951000	1.621414000
O	1.277197000	-1.102577000	2.610825000
C	0.392717000	-3.049647000	0.000157000
O	0.888448000	-4.093606000	0.001485000
C	0.675060000	-1.114642000	-1.622002000
O	1.277077000	-1.101933000	-2.611387000
Hg	0.614803000	1.227540000	0.000058000
Cl	1.397829000	3.536643000	0.000287000

19

[Mo(HgBr)(η^5-C_5H_5)(CO)$_3$]

Mo	-0.468632000	-1.278283000	-0.000197000
H	-2.497943000	-0.504330000	2.181344000
C	-2.489588000	-0.843282000	1.153789000
C	-2.480288000	-2.195791000	0.716614000
H	-2.537141000	1.073396000	-0.000018000
H	-2.497517000	-3.070618000	1.352776000
C	-2.480464000	-2.195758000	-0.716782000
H	-2.497789000	-3.070559000	-1.352979000
C	-2.489849000	-0.843242000	-1.153902000
H	-2.498408000	-0.504253000	-2.181444000
C	-2.502096000	-0.008289000	-0.000040000
C	0.672617000	-1.119451000	1.619964000
O	1.275215000	-1.105447000	2.609247000
C	0.389633000	-3.057340000	0.000157000
O	0.885310000	-4.101487000	0.001483000
C	0.672478000	-1.119145000	-1.620554000
O	1.275100000	-1.104788000	-2.609801000
Hg	0.616377000	1.223817000	0.000052000
Br	1.451764000	3.646192000	0.000294000

19

[Mo(HgI)(η^5-C_5H_5)(CO)$_3$]

Mo	-0.477959000	-1.287929000	-0.000210000
H	-2.508532000	-0.519310000	2.181366000
C	-2.500115000	-0.857970000	1.153703000
C	-2.489014000	-2.210340000	0.716595000
H	-2.549626000	1.059088000	-0.000029000
H	-2.504132000	-3.085253000	1.352712000
C	-2.489176000	-2.210306000	-0.716781000
H	-2.504381000	-3.085194000	-1.352932000
C	-2.500357000	-0.857933000	-1.153838000
H	-2.508957000	-0.519234000	-2.181487000
C	-2.513186000	-0.022535000	-0.000049000
C	0.664386000	-1.115593000	1.617071000
O	1.268233000	-1.094660000	2.606074000
C	0.390894000	-3.060738000	0.000146000
O	0.893012000	-4.102217000	0.001636000
C	0.664204000	-1.115238000	-1.617692000
O	1.268000000	-1.093998000	-2.606706000
Hg	0.614603000	1.221281000	0.000056000
I	1.580883000	3.779422000	0.000369000

19
[W(HgCl)(η⁵-C₅H₅)(CO)₃]

W	-0.462351000	-1.270751000	-0.000119000
H	-2.504475000	-0.494845000	2.181597000
C	-2.494171000	-0.834139000	1.154244000
C	-2.483124000	-2.188575000	0.716895000
H	-2.541456000	1.081638000	-0.000003000
H	-2.502415000	-3.062951000	1.353543000
C	-2.483235000	-2.188545000	-0.717020000
H	-2.502555000	-3.062893000	-1.353706000
C	-2.494341000	-0.834094000	-1.154310000
H	-2.504773000	-0.494759000	-2.181649000
C	-2.508649000	-0.000049000	-0.000021000
C	0.687275000	-1.126474000	1.626843000
O	1.291606000	-1.107491000	2.616777000
C	0.407934000	-3.056331000	0.000156000
O	0.904151000	-4.102102000	0.000689000
C	0.687216000	-1.126421000	-1.627146000
O	1.291513000	-1.107401000	-2.617095000
Hg	0.613059000	1.244646000	0.000078000
Cl	1.397571000	3.552878000	0.000249000

19
[W(HgBr)(η⁵-C₅H₅)(CO)₃]

W	-0.466094000	-1.279376000	-0.000139000
H	-2.507409000	-0.502070000	2.181611000
C	-2.497555000	-0.841285000	1.154230000
C	-2.488094000	-2.195646000	0.716904000
H	-2.542661000	1.074748000	0.000000000
H	-2.508229000	-3.070045000	1.353491000
C	-2.488217000	-2.195609000	-0.717055000
H	-2.508389000	-3.069974000	-1.353686000
C	-2.497743000	-0.841230000	-1.154309000
H	-2.507736000	-0.501966000	-2.181674000
C	-2.510930000	-0.006993000	-0.000024000
C	0.684669000	-1.130336000	1.625159000
O	1.289863000	-1.108804000	2.614763000
C	0.404715000	-3.064070000	0.000186000
O	0.900538000	-4.110167000	0.000864000
C	0.684585000	-1.130235000	-1.625524000
O	1.289725000	-1.108644000	-2.615152000
Hg	0.613508000	1.240968000	0.000077000
Br	1.454233000	3.662076000	0.000280000

19
[W(HgI)(η⁵-C₅H₅)(CO)₃]

W	-0.475060000	-1.289117000	-0.000199000
H	-2.517866000	-0.517223000	2.181608000
C	-2.507725000	-0.856152000	1.154128000
C	-2.496360000	-2.210391000	0.716845000
H	-2.555362000	1.060244000	-0.000005000
H	-2.514696000	-3.084925000	1.353317000
C	-2.496521000	-2.210341000	-0.717056000
H	-2.514912000	-3.084831000	-1.353588000
C	-2.507966000	-0.856080000	-1.154249000
H	-2.518280000	-0.517086000	-2.181707000
C	-2.521953000	-0.021440000	-0.000038000
C	0.676559000	-1.126060000	1.622057000
O	1.282836000	-1.096959000	2.611416000
C	0.405353000	-3.067728000	0.000263000
O	0.906235000	-4.111813000	0.001362000
C	0.676409000	-1.125803000	-1.622592000
O	1.282577000	-1.096563000	-2.612003000
Hg	0.613112000	1.238363000	0.000074000
I	1.582400000	3.795248000	0.000369000

17

$[Co(HgCl)(\eta^5\text{-}C_5H_5)(CO)_2]^+$

Co	-0.714981000	-1.297093000	-0.349873000
H	-2.036321000	-1.016408000	2.139577000
C	-2.211095000	-1.127407000	1.077187000
C	-2.350298000	-2.377827000	0.374594000
H	-2.359180000	0.991840000	0.361200000
H	-2.280906000	-3.362319000	0.818813000
C	-2.539366000	-2.088212000	-0.992962000
H	-2.639646000	-2.808994000	-1.794189000
C	-2.518801000	-0.655597000	-1.150389000
H	-2.617408000	-0.121124000	-2.086460000
C	-2.361441000	-0.067002000	0.137683000
C	0.505885000	-2.028024000	0.697689000
O	1.274216000	-2.527349000	1.375845000
C	0.146973000	-1.520082000	-1.875615000
O	0.684841000	-1.693108000	-2.865622000
Hg	0.540822000	0.844502000	-0.104296000
Cl	1.728334000	2.890193000	0.142051000

17

$[Co(HgBr)(\eta^5\text{-}C_5H_5)(CO)_2]^+$

Co	-0.719581000	-1.305307000	-0.350492000
H	-2.040693000	-1.022842000	2.138872000
C	-2.215656000	-1.133952000	1.076558000
C	-2.356721000	-2.384582000	0.373918000
H	-2.360514000	0.985337000	0.360371000
H	-2.287958000	-3.369191000	0.817905000
C	-2.545340000	-2.094744000	-0.993455000
H	-2.646310000	-2.815252000	-1.794834000
C	-2.522912000	-0.662027000	-1.150863000
H	-2.621209000	-0.127526000	-2.086937000
C	-2.364670000	-0.073513000	0.136902000
C	0.501678000	-2.034273000	0.697070000
O	1.270059000	-2.533636000	1.375437000
C	0.142770000	-1.526571000	-1.875720000
O	0.680198000	-1.699032000	-2.866324000
Hg	0.542119000	0.841539000	-0.104985000
Br	1.796369000	2.991562000	0.151808000

17

$[Co(HgI)(\eta^5\text{-}C_5H_5)(CO)_2]^+$

Co	-0.726567000	-1.316732000	-0.351748000
H	-2.047259000	-1.033124000	2.137444000
C	-2.222502000	-1.143841000	1.075142000
C	-2.366086000	-2.394766000	0.372837000
H	-2.364471000	0.975750000	0.359124000
H	-2.298393000	-3.379242000	0.817172000
C	-2.554498000	-2.104978000	-0.993989000
H	-2.656560000	-2.825195000	-1.795430000
C	-2.529563000	-0.671929000	-1.151521000
H	-2.627589000	-0.137700000	-2.087767000
C	-2.370255000	-0.083092000	0.135790000
C	0.496217000	-2.039746000	0.695646000
O	1.265452000	-2.538452000	1.374454000
C	0.137458000	-1.532985000	-1.875041000
O	0.675361000	-1.704771000	-2.866137000
Hg	0.542841000	0.839424000	-0.105791000
I	1.898043000	3.127370000	0.165049000

17

[Rh(HgCl)(η^5-C_5H_5)$(CO)_2$]$^+$

Rh	-0.629223000	-1.307359000	-0.363010000
H	-2.154688000	-1.024974000	2.156941000
C	-2.310038000	-1.135522000	1.091619000
C	-2.472639000	-2.386594000	0.388606000
H	-2.436702000	0.985026000	0.374262000
H	-2.428884000	-3.371206000	0.835472000
C	-2.660070000	-2.094724000	-0.979516000
H	-2.785732000	-2.813816000	-1.778534000
C	-2.615592000	-0.659900000	-1.139100000
H	-2.733578000	-0.123454000	-2.071583000
C	-2.464157000	-0.073579000	0.152432000
C	0.717580000	-2.073251000	0.730822000
O	1.499026000	-2.562629000	1.399354000
C	0.340030000	-1.544370000	-1.975243000
O	0.890622000	-1.713884000	-2.957649000
Hg	0.671277000	0.937243000	-0.106921000
Cl	1.824395000	2.998984000	0.147280000

17

[Rh(HgBr)(η^5-C_5H_5)$(CO)_2$]$^+$

Rh	-0.633665000	-1.317357000	-0.360776000
H	-2.162596000	-1.041003000	2.157876000
C	-2.316980000	-1.147247000	1.091974000
C	-2.481982000	-2.395664000	0.384716000
H	-2.438737000	0.976146000	0.382188000
H	-2.440878000	-3.381848000	0.828306000
C	-2.666887000	-2.098760000	-0.982419000
H	-2.792629000	-2.814622000	-1.784309000
C	-2.618331000	-0.663488000	-1.137081000
H	-2.733572000	-0.123752000	-2.068014000
C	-2.467996000	-0.081602000	0.156585000
C	0.713226000	-2.079562000	0.734318000
O	1.495065000	-2.567800000	1.403640000
C	0.339854000	-1.548370000	-1.970494000
O	0.893570000	-1.714407000	-2.952030000
Hg	0.668344000	0.935685000	-0.108847000
Br	1.895822000	3.099639000	0.129599000

17

[Rh(HgI)(η^5-C_5H_5)$(CO)_2$]$^+$

Rh	-0.638290000	-1.334585000	-0.361639000
H	-2.169218000	-1.055289000	2.157268000
C	-2.323427000	-1.159056000	1.091093000
C	-2.495877000	-2.405760000	0.382402000
H	-2.437141000	0.966056000	0.385037000
H	-2.460724000	-3.392614000	0.824907000
C	-2.679128000	-2.106187000	-0.983759000
H	-2.809336000	-2.820089000	-1.786638000
C	-2.622364000	-0.670879000	-1.136528000
H	-2.735712000	-0.129451000	-2.066715000
C	-2.469422000	-0.091194000	0.157630000
C	0.710177000	-2.088502000	0.734097000
O	1.494413000	-2.573331000	1.403990000
C	0.339369000	-1.556642000	-1.967973000
O	0.896757000	-1.718271000	-2.948788000
Hg	0.666152000	0.931309000	-0.110266000
I	1.985398000	3.240472000	0.131116000

17

$[Ir(HgCl)(\eta^5\text{-}C_5H_5)(CO)_2]^+$

Ir	-0.617792000	-1.301471000	-0.363574000
H	-2.183987000	-1.021073000	2.164511000
C	-2.330924000	-1.134678000	1.098611000
C	-2.474940000	-2.384992000	0.392737000
H	-2.450235000	0.983686000	0.375263000
H	-2.424959000	-3.370945000	0.836068000
C	-2.663471000	-2.092319000	-0.981254000
H	-2.782256000	-2.814328000	-1.778734000
C	-2.637553000	-0.658386000	-1.139774000
H	-2.764082000	-0.119911000	-2.069627000
C	-2.483513000	-0.075168000	0.154582000
C	0.732085000	-2.080650000	0.709388000
O	1.516539000	-2.580635000	1.371532000
C	0.359824000	-1.556276000	-1.962705000
O	0.915508000	-1.735739000	-2.943897000
Hg	0.697316000	0.959099000	-0.105830000
Cl	1.844068000	3.019776000	0.147936000

17

$[Ir(HgBr)(\eta^5\text{-}C_5H_5)(CO)_2]^+$

Ir	-0.622606000	-1.311644000	-0.362147000
H	-2.190894000	-1.035250000	2.165087000
C	-2.337262000	-1.145365000	1.098745000
C	-2.484130000	-2.393481000	0.389598000
H	-2.451831000	0.975368000	0.381464000
H	-2.436669000	-3.380742000	0.830226000
C	-2.670726000	-2.096686000	-0.983616000
H	-2.790045000	-2.816143000	-1.783302000
C	-2.640636000	-0.662454000	-1.138291000
H	-2.764864000	-0.121271000	-2.066884000
C	-2.486889000	-0.082781000	0.157754000
C	0.728152000	-2.086304000	0.711990000
O	1.513721000	-2.584087000	1.374911000
C	0.358997000	-1.561134000	-1.959053000
O	0.917804000	-1.737100000	-2.939400000
Hg	0.694010000	0.957067000	-0.108674000
Br	1.915495000	3.117997000	0.136824000

17

$[Ir(HgI)(\eta^5\text{-}C_5H_5)(CO)_2]^+$

Ir	-0.623453000	-1.336934000	-0.346274000
H	-2.210423000	-1.126163000	2.176082000
C	-2.350920000	-1.196173000	1.105463000
C	-2.510993000	-2.416813000	0.352661000
H	-2.428873000	0.950027000	0.464949000
H	-2.485561000	-3.419459000	0.758691000
C	-2.680376000	-2.067924000	-1.010318000
H	-2.802353000	-2.756647000	-1.836326000
C	-2.625496000	-0.630383000	-1.115177000
H	-2.734403000	-0.055293000	-2.025073000
C	-2.474859000	-0.098759000	0.202499000
C	0.721294000	-2.090598000	0.748129000
O	1.507578000	-2.575569000	1.420472000
C	0.373233000	-1.605687000	-1.928697000
O	0.942955000	-1.793922000	-2.901073000
Hg	0.688981000	0.953057000	-0.137267000
I	1.945298000	3.303229000	-0.023506000
